# A mutation in RNA polymerase imparts resistance to β-lactams by preventing dysregulation of amino acid and nucleotide metabolism

**DOI:** 10.1016/j.celrep.2025.115268

**Published:** 2025-02-04

**Authors:** Yesha Patel, John D. Helmann

**Affiliations:** 1 Department of Microbiology, Cornell University, Ithaca, NY 14853-8101, USA; 2 Lead contact

## Abstract

Resistance to diverse antibiotics can result from mutations in RNA polymerase (RNAP), but the underlying mechanisms remain poorly understood. In this study, we compare two *Bacillus subtilis* RNAP mutations: one in β′ (*rpoC* G1122D) that increases resistance to cefuroxime (CEF; a model β-lactam) and one in β (*rpoB* H482Y) that increases sensitivity. CEF resistance is mediated by a decrease in branched-chain amino acid (BCAA), methionine, and pyrimidine pathways. These same pathways are upregulated by CEF, and their derepression increases CEF sensitivity and antibiotic-induced production of reactive oxygen species. The CEF-resistant *rpoC* G1122D mutant evades these metabolic perturbations, and repression of the BCAA and pyrimidine pathways may function to restrict membrane biogenesis, which is beneficial when cell wall synthesis is impaired. These findings provide a vivid example of how RNAP mutations, which commonly arise in response to diverse selection conditions, can rewire cellular metabolism to enhance fitness.

## In brief

Patel and Helmann identify metabolic pathways that are dysregulated in RNAP mutant strains with altered β-lactam sensitivity. Genetic and physiological studies demonstrate that BCAA and Pyr synthesis are repressed in a β-lactam-resistant strain. Derepression of these pathways, genetically or by β-lactams, leads to ROS production and antibiotic sensitivity.

## Graphical Abstract

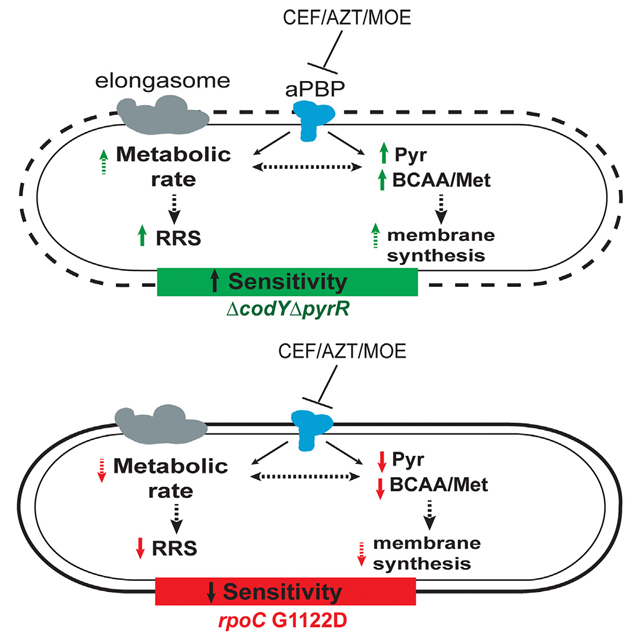

## INTRODUCTION

Motivated in part by the looming threat of the Second World War, Howard Florey, Ernst Chain, and their colleagues at Oxford worked to isolate the active ingredient responsible for the anti-biotic activity of the *Penicillium* fungus discovered a decade prior by Alexander Fleming.^1–3^ The subsequent optimization and scale-up of fermentation methods for production of penicillin, in large part in the United States,^4^ is a milestone in the history of antibiotics. To this day, penicillin and related compounds (the β-lactams) are among the most important tools for the clinical management of many infections.^5,6^

β-lactams inhibit transpeptidation by covalently modifying penicillin-binding proteins (PBPs),^7^ the key enzymes required for crosslinking the glycan strands that make up the peptidoglycan (PG) meshwork of the bacterial cell wall.^8,9^ β-lactam antibiotics are often bactericidal and kill cells when the weakened PG layer results in cell lysis.^10^ However, cell death can occur independent of lysis, perhaps due to metabolic depletion from futile cycling.^11^ Alternatively, β-lactams may have a bacteriostatic effect; for example, when cell integrity is stabilized by the outer membrane to prevent lysis.^11,12^ In select cases, cells may evade antibiotics to grow as wall-less cells (L-forms).^13^ In addition to their direct effects on wall synthesis, β-lactams also trigger metabolic perturbations that affect susceptibility,^14,15^ including the production of toxic reactive oxygen species (ROS).^16,17^

The emergence of β-lactam resistance presents a major challenge for their clinical use. The most widespread mechanisms rely on acquisition of new genes, such as those encoding β-lactam-degrading enzymes (β-lactamases)^18^ or low-affinity PBPs.^19^ In some organisms, β-lactam-insensitive L,D-transpeptidases allow a target-bypass mechanism.^20,21^ Even in the absence of specific defense mechanisms, bacteria often display an ability to defend against β-lactams and other cell wall-acting antibiotics through the activation of specific cell envelope stress response pathways.^22–24^ These intrinsic, adaptational mechanisms may facilitate survival and can increase the chances of high-level resistance emerging through mutations.^11^

RNA polymerase (RNAP) mutations are also frequently encountered in genetic studies of β-lactam resistance.^25–28^ However, how RNAP mutations confer resistance to β-lactams is not clear. In previous studies, we identified mutations in two essential core subunits of *Bacillus subtilis* RNAP, β and β′, which lead to altered sensitivity to cefuroxime (CEF), a β-lactam of the cephalosporin class.^29^ A mutation in *rpoC* that results in an altered β′ subunit (G1122D) confers CEF resistance (CEF^R^), which was partially attributed to the impact of the mutation on the relative activities of alternative s subunits that play a key role in β-lactam resistance.^29,30^ In another study, we observed that a common rifampicin resistance (RIF^R^) mutation in *rpoB* (H482Y) was CEF sensitive (CEF^S^).^31^ RIF inhibits RNAP, and resistance is attributed to *rpoB* mutations that inhibit the binding of RIF to the β subunit.^32^ The increased CEF^S^ of the *rpoB* H482Y strain was correlated with altered levels of UDP-GlcNAc, an amino sugar PG precursor, likely due to secondary effects on the transcriptome.^31^ This type of collateral sensitivity has also been seen in *Mycobacterium tuberculosis*, where evolution of resistance to one drug can lead to enhanced sensitivity to a different, unrelated drug.^33^

In this study, we exploit the divergent phenotypes of these *rpoB* and *rpoC* mutants against β-lactams to understand how alterations in cell physiology contribute to antibiotic resistance. By comparing changes in the transcriptome resulting from CEF^R^ and CEF^S^ mutations, we identified specific amino acid and pyrimidine biosynthesis pathways that were differentially affected by these two RNAP mutations. We further show that CEF treatment induces expression of these pathways and that the *rpoC* mutation that confers CEF^R^ impedes this induction. Genetic changes that derepress these pathways sensitize cells to CEF, which reveals a direct connection between CEF-induced alterations in cell metabolism and antibiotic susceptibility.

## RESULTS

### RNAP mutations modulate drug susceptibility

In previous studies, we reported a mutation in β′ (*rpoC* G1122D) that confers CEF^R^, a β-lactam highly active against *B. subtilis*.^29^ Here, we confirm this finding and additionally show that this strain has a collateral sensitivity to RIF (Table S1). In contrast, a common RIF^R^ mutation in β (*rpoB* H482Y) imparts a CEF^S^ phenotype,^31^ as also seen here (Table S1). CEF targets PBP1(*ponA*),^34^ the major class A PBP in *B. subtilis*, and Δ*ponA* mutants have CEF^R^.^35^ To test whether the *rpoC* G1122D mutation functions by reducing PBP1 expression, we compared the CEF^R^ of each single mutant with the *rpoC* G1122D Δ*ponA* double mutant. Since the double mutant had greater CEF^R^ than the single mutant (Table S1), we infer that the effect of the *rpoC* G1122D mutation is not due to loss of *ponA* expression.

**Figure 1.**
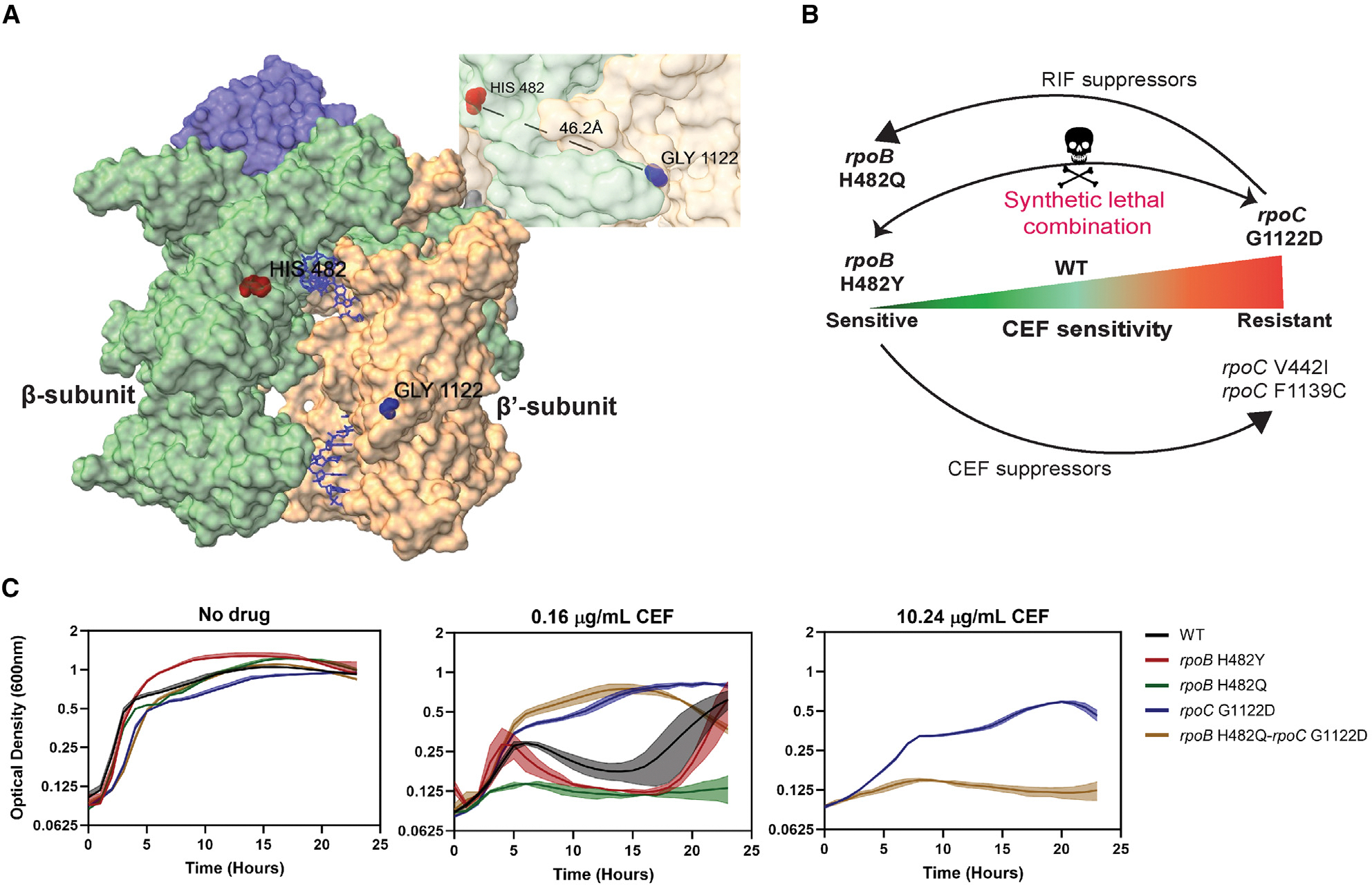
RNAP mutations alter drug susceptibilities (A) The β H482 residue (red) is a common site of rifampicin resistance (RIF^R^) mutations (equivalent to H526 in *E. coli* and H445 in *M. tuberculosis*), and the β′ G1122D substitution (blue) confers cefuroxime resistance (CEF^R^). The magnified image highlights the distance between these two residues. (B) Schematic of the amino acid substitutions in the *rpoB* H482Y (CEF^S^, left) and *rpoC* G1122D (CEF^R^, right) mutant strains. Several attempts to construct the *rpoB* H482Y-*rpoC* G1122D double mutant failed, indicating a synthetic lethal combination. Forward selection for RIF^R^ in the *rpoC* G11222D mutant resulted in the emergence of a H482Q mutation in the β subunit instead of the commonly found H482Y mutation. Forward selection for CEF^R^ in the *rpoB* H482Y mutant led to emergence of *rpoC* mutations (V442I and F1139C). (C) Growth of the different *rpoB* and *rpoC* mutants with CEF treatment. Left: the growth of the WT (HB26336), *rpoB* H482Y (HB26341), *rpoB* H482Q (HB28141), *rpoC* G1122D (HB26291), and *rpoB* H482Q-*rpoC* G1122D (HB26332) mutants in LB medium without any drug treatments. Center: comparison of their growth in the presence of 0.16 μg/mL of CEF. Right: the difference between the *rpoC* G1122D and *rpoB* H482Q-*rpoC* G1122D mutants at a high concentration of 10.24 μg/mL of CEF, where all other strains exhibit complete growth inhibition. *n* = 3. The data are plotted as an average; the shaded region represents the standard deviation between the replicates.

In the RNAP holoenzyme structure (PDB: 6WVJ), the β H482 and β′ G1122 residues are not at the subunit interface and do not contact one another (Figure 1A). To determine which of these mutations might be dominant, we sought to construct a double mutant. However, our attempts to generate an *rpoB* H482Y *rpoC* G1122D strain were unsuccessful. This incompatibility could be due to structural effects. However, these mutations, individually or together, were not predicted to have a destabilizing effect on RNAP (as determined using DDMut^36^). We therefore hypothesized that their incompatibility results from physiological perturbations caused by altered transcription.

Next, we employed forward genetics to explore how pre-existing mutations in RNAP influence the evolution of antibiotic resistance. By selecting for CEF^R^ in the *rpoB* H482Y (RIF^R^ CEF^S^) strain, we identified changes in *rpoC* leading to V442I (G1324A) or F1139C (T3416G) substitutions (Figure 1B), with no other changes detected. Next, we selected for RIF^R^ in the *rpoC* G1122D (CEF^R^ RIF^S^) strain (Figure 1B). Although many different mutations in the RIF^R^-determining region of *rpoB* can confer RIF^R^,^37^ the frequency of RIF^R^ suppressors in the *rpoC* mutant was unexpectedly low (Figure S1), and we only recovered two colonies from independent cultures. Both encode the same amino acid change (H482Q) at the position of the incompatible H482Y substitution (Table S1). The absence of other suppressors suggests that the pre-existing *rpoC* G1122D mutation may be incompatible with other commonly arising RIF^R^ mutations and is uniquely compatible with the H482Q change (Figure 1B). The *rpoB* H482Q mutation confers RIF^R^ both alone and in combination with *rpoC* G1122D (Table S1).

In liquid medium, the mutants did not have drastic differences in their growth in the absence of antibiotic (Figure 1C, left). As expected, both the RIF^R^ CEF^S^ *rpoB* mutants had growth defects relative to the wild type (WT) at 0.16 μg/mL CEF, although their growth patterns differed (Figure 1C, center), and the *rpoB* H482Q-*rpoC* G1122D double mutant had CEF^R^. However, only the *rpoC* G1122D mutant, and not the *rpoB* H482Q-*rpoC* G1122D double mutant, was able to grow at 10.24 μg/mL CEF (Figure 1C, right). These differences in minimum inhibitory concentration (MIC) and growth suggest that these RNAP mutations have distinct physiological consequences that either increase or decrease sensitivity to β-lactams.

### The CEF^R^ *rpoC* G1122D mutant has reduced expression of specific metabolic pathways

**Figure 2.**
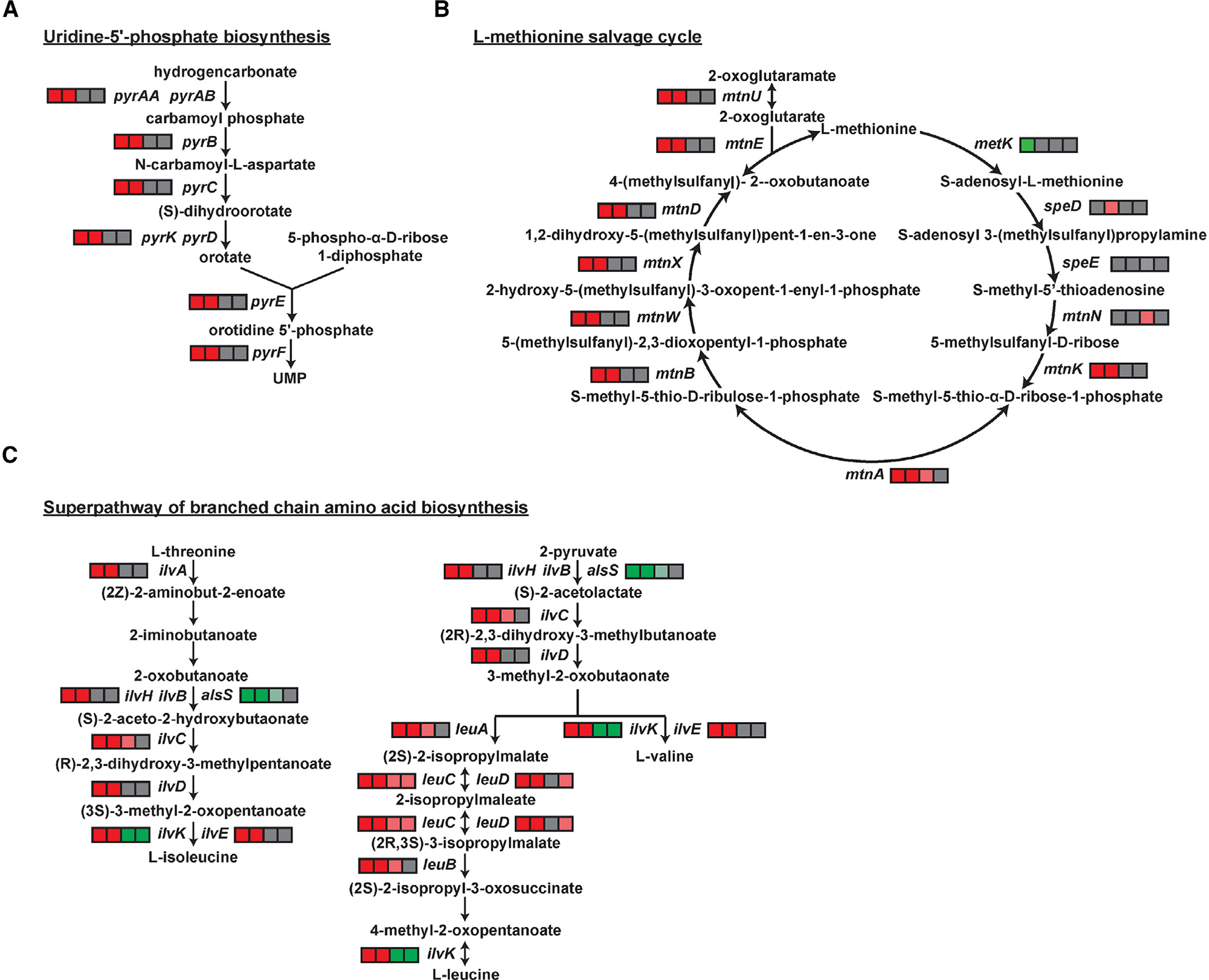
RNAP mutations alter the cellular transcriptome The highest pathway perturbation scores correspond to (A) uridine-5′-phosphate (UMP) biosynthesis/pyrimidine (Pyr) synthesis, (B) L-methionine salvage (Met), and (C) branched-chain amino acid (BCAA) metabolism. Changes in expression for each gene of these pathways are represented by squares using a scale from pink to red for reduced expression, green for increased expression, and gray for no significant change. The four squares represent the strains in the following order from left to right: *rpoC* G1122D, *rpoB* H482Q-*rpoC* G1122D, *rpoB* H482Y, and *rpoB* H482Q. The expression values are shown in Table S2.

Mutations in RNAP can lead to significant changes in the cellular transcriptome.^28^ To identify transcriptional changes that might account for the differences in β-lactam sensitivity (Table S1), we used RNA sequencing (RNA-seq) to identify differentially expressed genes in the mutants compared to the WT (Figure S2). Differentially expressed genes were visualized using volcano plots and mapped onto metabolic pathways using BsubCyc (https://bsubcyc.org/) (Figure S2). We first focused on pathways that were significantly altered in the two CEF^R^ strains (*rpoC* G1122D and *rpoB* H482Q-*rpoC* G1122D double mutant) but not in the two CEF^S^ strains (*rpoB* H482Q and H482Y). The pathways with the highest pathway perturbation scores included pyrimidine (Pyr) biosynthesis (Figure 2A), the methionine (Met) salvage cycle (Figure 2B), and branched-chain amino acid (BCAA) uptake and metabolism (Figure 2C). Expression of nearly all genes in these pathways was significantly reduced in the CEF^R^ but not in the CEF^S^ strains (Table S2).

### CEF triggers induction of the BCAA, Met, and Pyr pathways in the WT but not in the *rpoC* G1122D mutant

**Figure 3.**
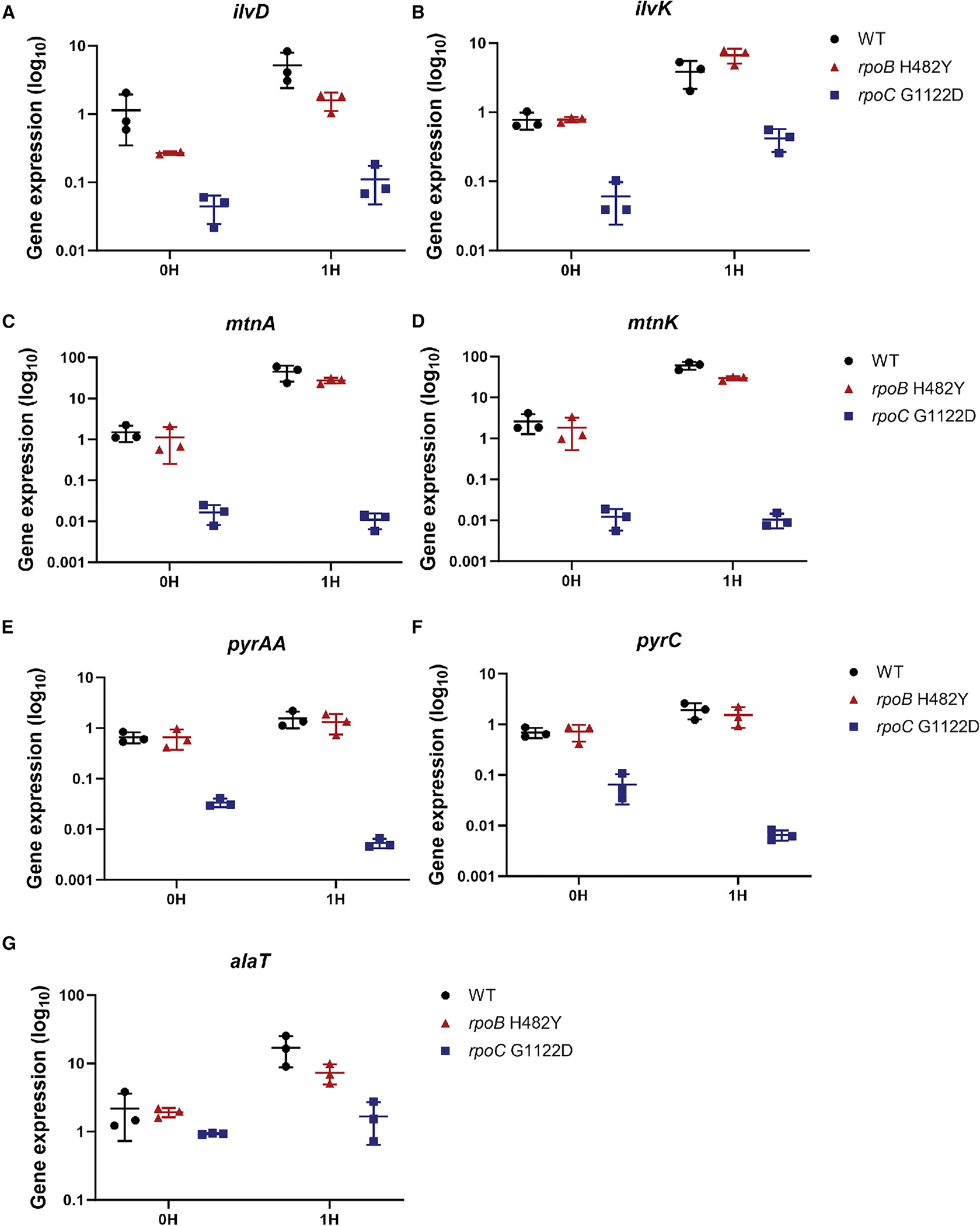
CEF treatment induces genes of BCAA synthesis, Met salvage, and Pyr synthesis Shown is real-time PCR quantitation of (A) *ilvD* and (B) *ilvK* representing BCAA synthesis, (C) *mtnA* and (D) *mtnK* representing Met salvage, (E) *pyrAA* and (F) *pyrC* representing Pyr biosynthesis, and (G) *alaT* representing alanine synthesis (a control biosynthetic gene). Expression of these genes was quantified in the WT (HB26336), *rpoB* H482Y (HB26341), and *rpoC* G1122D (HB26291) mutant before and 1 h after treatment with 0.64 μg/mL of CEF. Gene expression values are plotted on a log_10_ scale after normalization to *gyrA* as an internal control. *n* = 3. Each dot represents the individual value, and error bars represent the standard deviation.

We used real-time PCR to compare the response of selected genes in the BCAA (*ilvD* and *ilvK)*, Met (*mtnA* and *mtnK*), and Pyr (*pyrC* and *pyrAA*) pathways to CEF in the WT, *rpoB* H482Y (CEF^S^), and *rpoC* G1122D (CEF^R^) strains. A sub-inhibitory concentration of CEF strongly induced Met genes in both WT and *rpoB* H482Y cells, with more modest induction noted for the BCAA and Pyr genes (Figure 3). As expected, expression of all selected genes (except the control gene *alaT*) was dramatically reduced in the CEF^R^ *rpoC* G1122D mutant compared to the WT and the CEF^S^ *rpoB* H482Y mutant (Figure 3), validating the lower expression observed by RNA-seq (Figure 2). Strikingly, in the *rpoC* mutant, these genes were no longer strongly induced by CEF treatment (Figure 3). These results indicate that CEF elevates the expression of the BCAA, Met, and Pyr pathways and that this induction is selectively lost in the *rpoC* G1122D mutant.

### Supplemental amino acids do not increase CEF^S^ of WT cells

**Figure 4.**
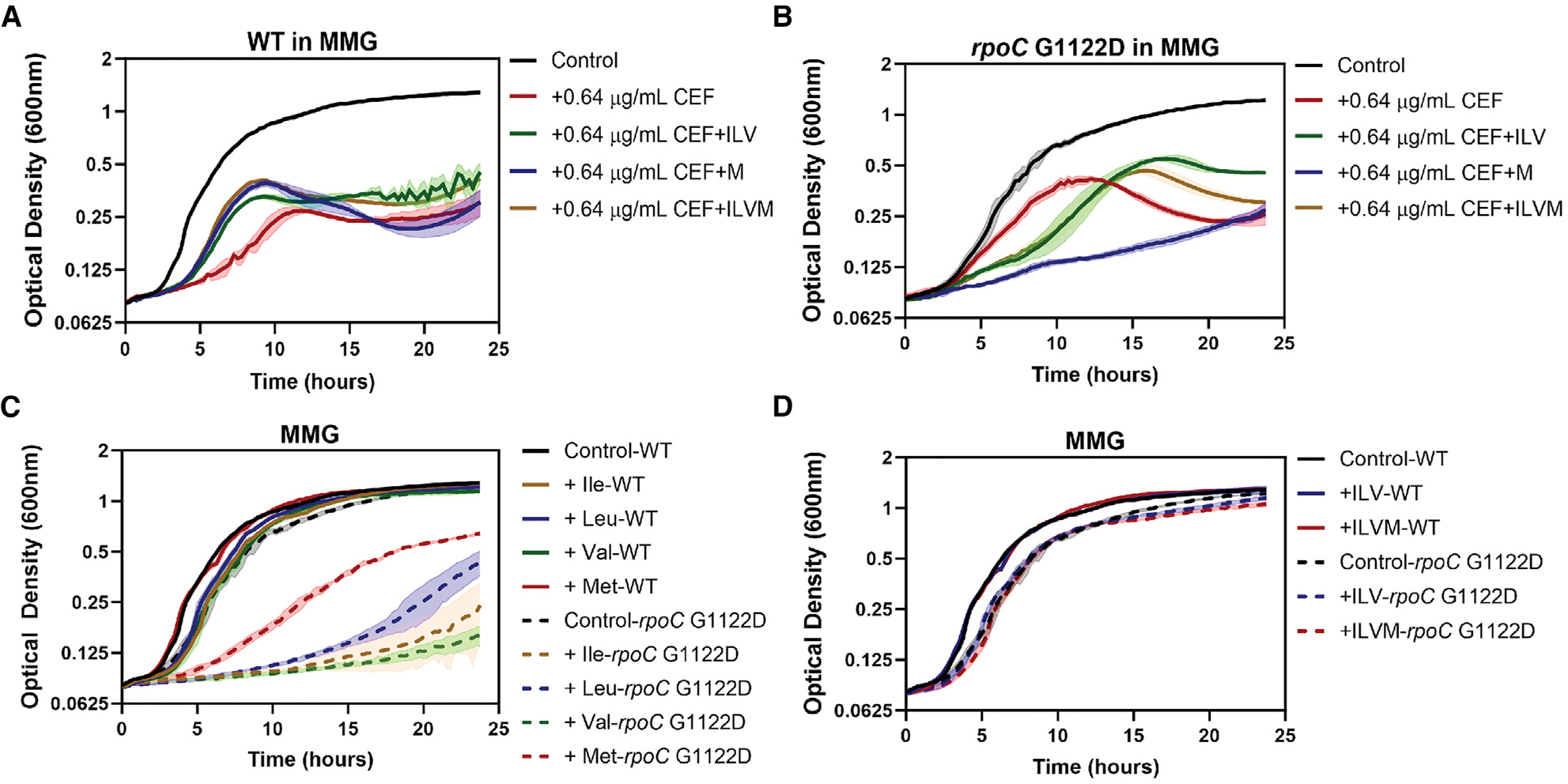
Effects of BCAA and Met supplementation on CEF^S^ of WT and *rpoC* G1122D mutants (A) Growth of WT (HB26336) in minimal medium with glucose (MMG) supplemented with the combination of 2 mg/mL of Ile, Leu, and Val (ILV) and/or Met (M) in the presence of 0.64 μg/mL CEF. (B) Growth of the *rpoC* G1122D (HB26291) mutant in MMG supplemented with the combination of 2 mg/mL of ILV and/or Met in the presence of 0.64 μg/mL CEF. (C) Effect of individual Ile, Leu, Val, and Met on the growth of the WT and *rpoC* G1122D mutant in MMG. (D) Effect of the combination of ILV and M on the WT and *rpoC* G1122D mutant in MMG. *n* = 3. The data are plotted as an average; the shaded region represents the standard deviation between the replicates.

Induction of BCAA and Met biosynthesis genes may lead to an increase in the levels of amino acids in the cells, and we hypothesized that these increased pools may be deleterious to the cell. However, amendment of glucose-containing minimal medium (MMG) with BCAAs (isoleucine, leucine, and valine; ILV) and/or Met (M) did not increase, but rather reduced, the CEF^S^ of WT cells (Figures 4A and S3A). This suggests that elevated levels of BCAA (ILV) or Met (M) do not by themselves confer CEF^S^. In contrast, high levels of ILV and/or M do increase CEF^S^ of the *rpoC* G1122D mutant (Figure 4B), perhaps because this strain has altered regulation of the corresponding biosynthetic pathways. Indeed, growth of the *rpoC* G1122D mutant, but not of the WT, was unexpectedly inhibited by high concentrations of the single amino acids Ile, Leu, Val, or Met (Figure 4C) but not by ILV (or ILVM) supplied in combination (Figure 4D). In contrast, supplemental Ala, Arg, Glu, Gln, His, and Pro did not significantly impair growth of either the WT (Figure S3B, left) or the *rpoC* G1122D mutant (Figure S3B, right) with or without CEF. Supplemental Ser did impair growth (Figures S3D and S3F), consistent with its known toxicity,^38^ and Thr, through its interference with BCAA synthesis^39,40^ inhibited growth and increased CEF^S^ for the *rpoC* G1122D mutant (Figure S3E) but not of the WT (Figure S3C). We conclude that supplemental amino acids do not by themselves confer CEF^S^ to the WT and that the reduced expression of amino acid biosynthesis genes in the *rpoC* G1122D mutant is correlated with growth sensitivity when cells are exposed to unbalanced pools of amino acids, possibly due to altered regulation.

### Derepression of the CodY regulon contributes to CEF^S^

Induction of ILVM synthesis genes by CEF in the WT strain (Figure 3) and their low and non-inducible expression in the CEF^R^ *rpoC* G1122D mutant (Figure 2) suggest a correlation between induction of ILVM synthesis genes and CEF^S^. Consistently, supplemental ILVM, which is predicted to repress these pathways, increased CEF^R^ in WT cells (Figure 4A). BCAA synthesis and up-take are regulated by the global regulator CodY, which is activated by BCAAs to bind DNA and repress gene expression.^41^

**Figure 5.**
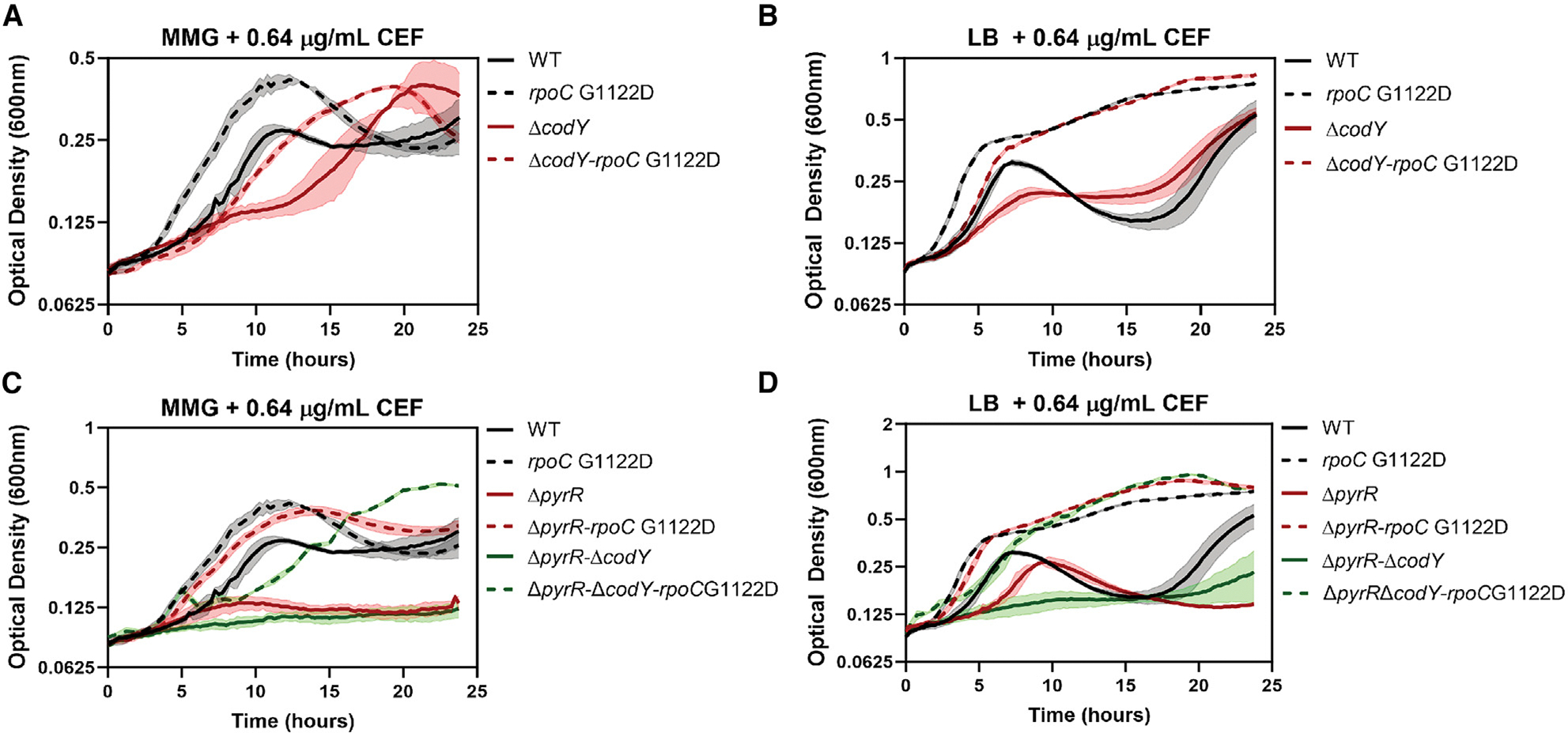
Derepression of BCAA and/or Pyr synthesis increases CEF^S^ (A and B) Growth of the WT (HB26336), *rpoC* G1122D (HB26291), Δ*codY* (HB28306) and, Δ*codY-rpoC* G1122D double mutant (HB28314) in (A) MMG with 0.64 μg/mL CEF and (B) LB with 0.64 μg/mL of CEF. (C and D) Growth of the WT, *rpoC* G1122D, Δ*pyrR* (HB28302), Δ*pyrR-rpoC* G1122D (HB28309), Δ*pyrR*Δ*codY* (HB28320), and Δ*pyrR*D*codY-rpoC* G1122D mutant (HB28380) in (C) MMG with 0. 64 μg/mL of CEF and (D) LB with 0.64 μg/mL of CEF. *n* = 3. The data is plotted as an average; the shaded region represents the standard deviation between the replicates.

We used a Δ*codY* mutant to explore the consequences of derepression of BCAA genes on CEF^R^. The Δ*codY* strain grows as well as the WT (Figure S4A) but had a 2-fold reduction in CEF MIC (Table S1) and increased CEF^S^ in both MMG (Figure 5A) and LB (Figure 5B). Conversely, WT cells with ectopic induction of CodY from an IPTG-inducible promoter (P*_hyspac_*) had CEF^R^ and were able to grow with 10.24 μg/mL CEF (Figure S4C). In the CEF^R^ *rpoC* G1122D mutant, introduction of the Δ*codY* mutation also increased CEF^S^ in MMG (Figure 5A), although there was little effect in LB (Figure 5B), with the double mutant still retaining 7-fold higher CEF^R^ compared to the WT (Table S1). These results suggest that the effect of *rpoC* G1122D on CEF^R^ is partially, but not entirely, mediated by CodY. We also observed that the Δ*codY*-*rpoC* G1122D strain was no longer inhibited by I, L, V, or M when grown in MMG (Figure S4B). Since the *rpoC* strain maintains a low expression level of BCAA and Met genes, we hypothesize that further repression by individual supplemental amino acids (I, L, V, or M) may inhibit growth (Figure 4C) and that Δ*codY* alleviates this repression. In addition to BCAAs, CodY is also regulated by guanosine triphosphate (GTP).^42^ Deletion of *relA*, a GTP pyrophosphokinase that synthesizes (p) ppGpp, may increase GTP levels,^43^ leading to increased CodY binding.^44^ The Δ*relA* strains behaves similarly as the strain with P*_hyspac_*-*codY* (Figure S4C) when grown with 10.24 μg/mL of CEF (Figure S4D). Collectively, reduced expression of CodY-regulated genes by amendment with BCAAs, by CodY induction (P*_hyspac_*-*codY*), in Δ*relA*, and in *rpoC* G1122D all lead to CEF^R^.

We next sought to understand why derepression of the CodY regulon contributes to CEF^S^. CodY also regulates the levels of σ^D^,^45^ which controls the expression of several autolytic enzymes (*lytC*, *lytD*, and *lytF*)^46,47^ that are known to affect sensitivity to cell wall-acting antibiotics.^48^ Deletion of *sigD*, *lytC*, *lytD*, and *lytF* led to increased CEF^R^ both in the WT and in the Δ*codY* mutant (Figure S4E). However, none of these genes were seen to be reduced in expression in the CEF^R^ *rpoC* G1122D mutant in our RNA-seq data. Further, σ^D^ was induced after CEF treatment in the WT and in the *rpoC* mutant, unlike the other CodY-regulated genes (Figure S4F). Thus, although these genes had a significant impact on CEF^R^, their expression was not altered by the *rpoC* mutation. These findings reveal that the *rpoC* G1122D mutation only affects a subset of the CodY regulon.

### Derepression of the PyrR regulon contributes to CEF^S^

In addition to the BCAA and Met pathways, expression of *pyr* genes was highly reduced in the *rpoC* G1122D mutant (Figure 2A). The *pyr* genes are under control of the RNA-binding regulatory protein PyrR.^49^ When Pyrs are in excess, they bind to PyrR, which then engages with *pyr* mRNA, facilitating transcription termination prior to gene synthesis.^50,51^ We therefore wished to test whether the reduced expression of *pyr* genes in the *rpoC* G1122D mutant was mediated by PyrR and whether this regulation is important for CEF^R^. A *pyrR* deletion mutation (Δ*pyrR*) leads to high level expression of the Pyr pathway.^51^ Like Δ*codY*, Δ*pyrR* also increased the CEF^S^ of the WT (Figures 5C and 5D) with a 3-fold reduction in the MIC (Table S1). However, the Δ*pyrR rpoC* G1122D double mutant was similar to the *rpoC* G1122D single mutant (Table S1), suggesting that the reduced expression of the Pyr pathway by the *rpoC* G1122D mutation does not depend on PyrR. Although derepression of the Pyr pathway increases WT CEF^S^, we were unable to obtain this same effect simply by Pyr supplementation (50 μg/mL of uracil or uridine) to MMG (Figures S5A and S5B).

Strikingly, the double deletion of *codY* and *pyrR* had an additive effect with greatly increased CEF^S^ in both LB and MMG (Figures 5C and 5D) and a 27-fold lower MIC than the WT (Table S1). In the highly CEF^S^ Δ*pyrR*Δ*codY* background, introduction of the *rpoC* G1122D mutation still led to a large increase in CEF^R^ (Table S1). In sum, these results suggest that CEF induces both the BCAA and Pyr pathways (Figure 3), that mutations (Δ*pyrR,* Δ*codY*) that derepress these pathways significantly increase CEF^S^ (Figure 5; Table S1), and that the *rpoC* G1122D-mediated CEF^R^ is correlated with reduced BCAA and Pyr expression that does not rely on increased activity of these regulatory proteins.

### The *rpoC* G1122D mutant is resistant to antibiotics that target class A PBPs

**Figure 6.**
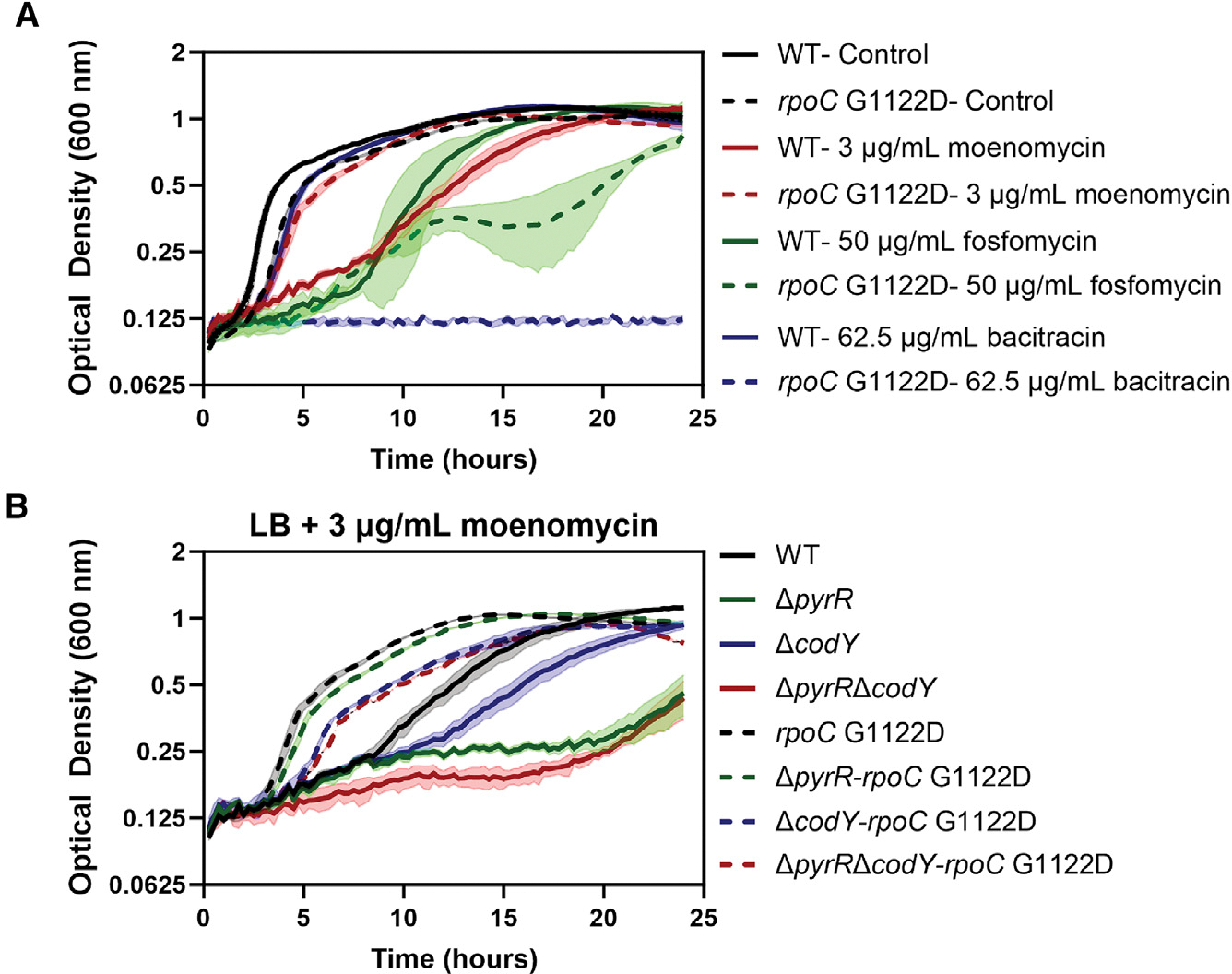
*rpoC* G1122D mutation imparts resistance against PBP1 inhibitors (A) Effect of antibiotics (dashed lines) on growth of the WT (HB26336) and *rpoC* G1122D (HB26291) mutant. Cells were treated with 3 μg/mL of MOE, 62.5 μg/mL of BAC, or 50 μg/mL of FOS as indicated. (B) Growth of *pyrR* (HB28302) and/or *codY* (HB28320/HB28306) deletion in the WT and *rpoC* G1122D mutation on treatment with 3 μg/mL of MOE. *n* = 3. The data are plotted as an average; the shaded region represents the standard deviation between the replicates.

We next evaluated whether the *rpoC* G1122D mutant had increased resistance against other antibiotics targeting PG synthesis, including moenomycin (MOE), fosfomycin (FOS), and bacitracin (BAC) as well as the monocyclic β-lactam aztreonam (AZT). MOE is a phosphoglycolipid that inhibits class A PBPs but targets the transglycosylase activity rather than the transpeptidase activity targeted by β-lactams.^52^ FOS inhibits the synthesis of PG amino sugar precursors,^53^ while BAC binds to undecaprenyl pyrophosphate, preventing its recycling.^54^ The *rpoC* G1122D mutant was more resistant to AZT (Figure S6A) and MOE but more sensitive to FOS and BAC (Figure 6A). The *pyrR* and *codY* deletions also increased the MOE^S^ of WT (but not the *rpoC* mutant), with a modest additivity observed for the Δ*pyrR*Δ*codY* double mutant (Figure 6B). In conclusion, the *rpoC* G1122D mutant was resistant to three different antibiotics that all target class A PBPs (CEF, AZT, and MOE) but was not resistant to β-lactams inhibiting other PBPs (Table S4) or other PG antibiotics (Figure 6A).

In gram-negative bacteria, the β-lactam mecillinam inhibits class A PBPs and has been postulated to lead to futile energy consumption due to increase in amino acid levels and protein synthesis.^15,55^ Inhibiting protein synthesis with sub-MIC levels of chloramphenicol mitigated the lytic effects of mecillinam. To test whether induction of the BCAA/Met pathways acts by a similar mechanism, we tested the effect of chloramphenicol on CEF-treated WT cells. However, sub-MIC chloramphenicol failed to enhance growth in the presence of CEF (Figure S6B). Thus, the CEF-dependent alterations in metabolism that contribute to CEF^S^ may be distinct from the effects in gram-negative bacteria.

### Repression of BCAA and Pyr metabolism may alter cell envelope synthesis

**Figure 7.**
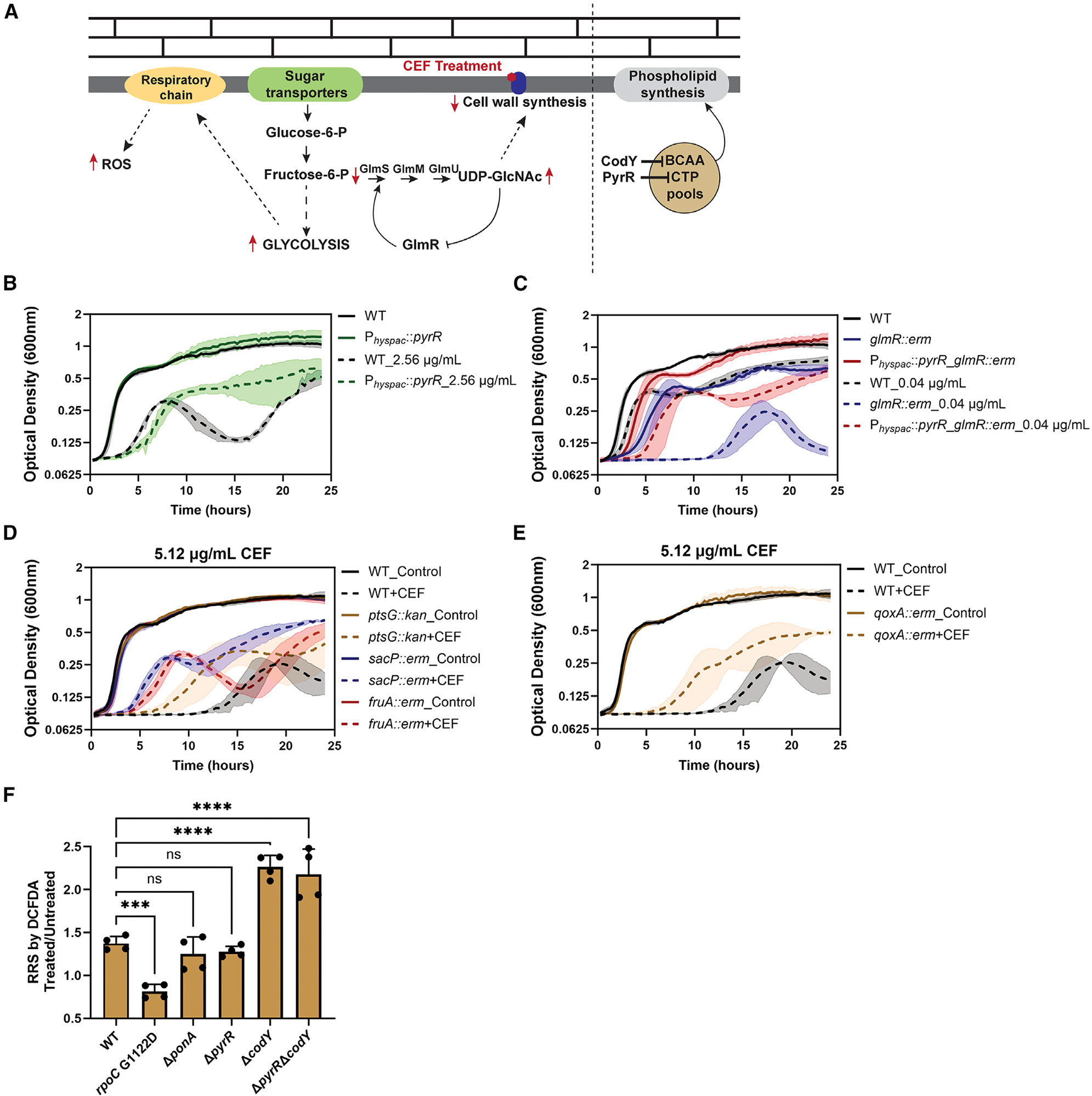
Induction of PyrR and mutations that alter central metabolic pathways increase CEF^R^ (A) Model illustrating the effects of BCAA and CTP levels on membrane synthesis (right). Both BCAAs and CTP are precursors in phospholipid synthesis, a key component of cell membranes. Repression of the BCAA and Pyr synthesis pathways by CodY and PyrR may help reduce membrane synthesis. Inhibition of cell wall synthesis by CEF increases the level of UDP-GlcNAc, the PG precursor. UDP-GlcNAc binds with GlmR to prevent GlmR-mediated activation of GlmS, which lowers the flux of sugars into cell wall synthesis (left). This results in increased flux through glycolysis, which can result in ROS production and cell lysis. The dotted lines represent multiple pathways that culminate into the mentioned end product. Arrows in red represent the changes in the levels of the intermediates. (B) The growth of WT (HB26336) and P_*hyspac*_::*pyrR* (HB28607) in LB on treatment with 2.56 μg/mL CEF and 1 mM IPTG supplemented for *pyrR* induction. (C) Growth of the WT (HB26336), *glmR*::*erm* (HB28007), and P_*hyspac*_::*pyrR-glmR*::*erm* (HB28628) double mutant in LB supplemented with 0.04 μg/mL CEF and 1 mM IPTG for *pyrR* induction. (D) Growth of the WT (HB26336) and mutant strains with reduced glycolytic flux due to loss of sugar-specific transporters for glucose (*ptsG*, HB28615), sucrose (*sacP*, HB28596), and fructose (*fruA*, HB28597) monitored in LB medium supplemented with 5.12 μg/mL CEF. (E) Growth of the WT (HB26336) and *qoxA*::*erm* (HB28617) strains in LB supplemented with 5.12 μg/mL CEF. For all growth curves, *n* = 3. The data are plotted as an average; the shaded region represents the standard deviation between the replicates. (F) The effect of CEF treatment on the production of reactive radical species (RRS) detected using DCFDA. A modest and consistent increase in RRS is seen in the WT (HB26336), Δ*ponA* (HB25950), and Δ*pyrR* (HB28302) strains. This increase in RRS is absent in the *rpoC* G1122D mutant but enhanced in the Δ*codY* (HB28306) and Δ*pyrR*Δ*codY* (HB28320) mutants. CEF (10.24 μg/mL) was added for 1 h, and all cells retained viability under these conditions. Values for each replicate are represented as independent dots (*n* = 4; error bars represent the standard deviation). One-way ANOVA was done to test the significance between the strains. ***p = 0.0006, ****p < 0.0001. ns, non-significant difference.

We hypothesized that the *rpoC* G1122D mutation might confer CEF^R^ by altering the synthesis of the cell envelope. BCAAs are precursors of branched-chain fatty acids that are major constituents of membrane phospholipids, and the Pyr nucleotide CTP is required for phosphatidate cytidylyltransferase, an essential enzyme for phospholipid synthesis^56^ (Figure 7A). Previously, we have demonstrated that cells limited for PG synthesis (Δ*rasp*Δ*ponA*) can be rescued by mutations that reduce fatty acid synthesis (affecting the acetyl-coenzyme A synthase or the FapR repressor).^57^ Since CEF also limits PG synthesis, we hypothesize that elevated activity of PyrR, and the consequent reduction in CTP levels, benefits cells by reducing membrane biogenesis. In support of this idea, induction of PyrR led to a marked reduction in cell lysis, which is typically seen in midexponential-phase cultures treated with 2.56 μg/mL CEF (Figure 7B). Induction of CodY also led to a striking CEF^R^ phenotype (Figure S4C), consistent with the important role of BCAAs as precursors for the synthesis of fatty acids and membrane lipids.

Reduced Pyr levels might also affect PG synthesis. Uridine diphosphate N-acetylglucosamine (UDP-GlcNAc) antagonizes GlmR (Figure 7A), an activator that allosterically increases flux of sugars through the branchpoint enzyme GlmS.^58,59^ In *glmR* mutants, flux of carbon into PG is restricted, and cells display a very long lag when challenged with CEF at low levels (0.04 μg/mL). A reduction of Pyr synthesis might increase the flux of sugars into PG synthesis by alleviating the inhibitory effect of UDP-GlcNAc on GlmR. However, repression of Pyr synthesis greatly improved growth even in a *glmR* null mutant, as seen in the P*_hyspac_*::*pyrR glmR::erm* strain treated with 0.04 μg/mL CEF (Figure 7C). Since repression of Pyr synthesis (by induction of PyrR) still increased CEF^R^ in a *glmR* null mutant, alteration of GlmR activity by UDP-GlcNAc cannot be the major effect of altering Pyr pools.

### Reduced flux through central carbon metabolism imparts CEF^R^

Several studies have linked the actions of bacteriostatic antibiotics to alterations of cell metabolism that, by mechanisms not fully resolved, can lead to increases in the production of ROS.^59^ For example, reducing metabolic flux through glycolysis and the respiratory chain^60^ promotes the formation of L-forms (cells lacking PG) in *B. subtilis* (Figure 7A). Similarly, we suggest that inhibition of PG synthesis by CEF may lead to increased glycolytic flux, respiratory activity, and production of ROS. Inspection of our RNA-seq data revealed that the *rpoC* G1122D mutant had reduced expression of sugar transporters for glucose (*ptsG)*, fructose (*fruA*), and sucrose (*sacP)* (Table S5). Mutant strains lacking any of these transporters, or lacking subunit II of the major *aa_3_* quinol oxidase (*qoxA*), displayed increased CEF^R^ (Figures 7D and 7E). We also observed increased repression of the Rex-controlled *cyd* genes and genes involved in anaerobic nitrate respiration (Table S5). This is reminiscent of β-lactam resistance in methicillin-resistant *Staphylococcus aureus*, in which mutations in *rpoB* and *rpoC* prevented the up-regulation of Rex-controlled anaerobic metabolism genes.^27,61^

### Production of RRS is correlated with CEF^S^

CEF treatment leads to elevated expression of the BCAA, Met, and Pyr pathways (Figure 3) and increased glycolytic flux,^62^ which may trigger increased ROS production.^17,60,63^ We hypothesized that the *rpoC* G1122D mutant (in which these pathways are repressed) might lack antibiotic-induced production of ROS. Here, we used the fluorescent probe 2’,7’-dichlorodihydrofluorescein diacetate (DCFDA)^64^ to monitor the production of ROS and other reactive radical species (RRS). Treatment of WT cells with 10.24 μg/mL CEF for 1 h increased the levels of RRS by ~40% as measured with DCFDA (Figure 7F). CEF also increased RRS in the Δ*ponA* mutant (Figure 7F), suggesting that RRS production is not only linked to PBP1 inhibition. RRS production was significantly higher in the Δ*codY* and Δ*pyrR*Δ*codY* deletion mutants (but not in the Δ*pyrR* mutant). Consistently, in the *rpoC* G1122D mutant, which does not induce the CodY regulon, there was no increase in RRS levels after CEF treatment. Although the *rpoC* G1122D mutant had decreased RRS production upon CEF treatment, this strain is still susceptible to ROS (H_2_O_2_, cumene hydroperoxide) and the superoxide generator paraquat (Figure S7A–S7C). This sensitivity is correlated with a decrease in expression of both KatA and AhpCF (Figure S7D). We conclude that the *rpoC* mutant is not resistant to oxidants in general.

## DISCUSSION

We used the model gram-positive bacterium *B. subtilis* to gain insights into intrinsic and acquired resistance mechanisms for β-lactams and other cell envelope-acting antibiotics.^22^ Intrinsic (or basal) resistance relies on specific stress responses. For example, PG synthesis inhibitors induce the σ^M^ regulon, which includes many of the enzymes required for cell wall synthesis,^22,65^ and this provides intrinsic resistance to CEF, MOE, and other cell envelope-active compounds.^30,66^ Acquired antibiotic resistance results from genetic changes. For example, CEF^R^ can arise from *gdpP* mutations that inactivate the major hydrolase that degrades cyclic di-AMP,^30^ as seen also in *Staphylococcus aureus*.^67–69^ Since β-lactam-mediated cell lysis may be driven by extrusion of the cell membrane-bounded cytosol through weak spots in the cell wall,^10^ high c-di-AMP may confer tolerance by reducing cell turgor.^70–72^ Consistently, mutations that decrease cell membrane synthesis are also able to rescue cells with a reduced capacity for PG synthesis.^57^

The wide availability of cost-effective whole-genome sequencing has revolutionized our ability to follow bacterial evolution both in the laboratory and in clinical settings. In some cases, a resistance mechanism can be inferred easily, including mutations that affect the antibiotic target, alter the cell envelope to reduce permeation, or increase efflux. Mutations in RNAP are frequently associated with β-lactam resistance in diverse organisms,^25–28,73^ but their mechanism of action is often complex and puzzling. Indeed, RNAP mutations arise frequently in adaptive laboratory evolution experiments,^74^ with changes often targeting conserved residues.^75^

Here, we took advantage of mutations in the core subunits of *B. subtilis* RNAP that have global impacts on the transcriptome to reveal metabolic pathways that correlate with CEF^S^. The *rpoC* G1122D mutation, which significantly increases CEF^R^,^29^ greatly reduces expression of the BCAA, Pyr, and Met synthesis pathways both in the absence and presence of antibiotic stress (Figures 2 and 3). Additionally, by remodeling the transcriptome to reduce expression of sugar importers, the *rpoC* G1122D mutation may reduce flux through glycolysis and, thereby, the level of CEF-induced RRS production (Figure 7). In other systems, PBP inhibition has also been linked to the production of ROS,^62,76,77^ although the nature of the RRS produced and their role in sensitivity have been debated.^78,79^

Exposure of WT cells to CEF leads to a striking transcriptional induction of many of these same pathways (Figure 3). We suggest that this induction contributes to CEF^S^. Genetic studies reveal that derepression of the CodY (BCAA synthesis) and PyrR (Pyr synthesis) regulons increases sensitivity to CEF (Figure 5; Table S1) and other class A PBP inhibitors (MOE and AZT; Figures 6 and S6), whereas induction of these regulators increases CEF^R^. Thus, these repressors are part of the intrinsic resistome. Since introduction of *rpoC* G1122D confers resistance even in a Δ*pyrR*Δ*codY* mutant, we suggest that changes in gene expression are due to the altered RNAP rather than a change in activity of the regulators. Altered regulation of the BCAA and Pyr synthesis pathways likely affects membrane biogenesis, which provides a plausible mechanism linking metabolic remodeling with CEF^R^.^57^

In summary, we demonstrate that the maladaptive transcriptional derepression of the CodY and PyrR regulons by CEF increases antibiotic sensitivity in WT cells. An RNAP mutation that impedes this transcriptional response can prevent this maladaptive change and increase CEF^R^. The CodY and PyrR regulons are widely conserved in gram-positive bacteria, and it is therefore likely that the principles elucidated here may be relevant to important human pathogens. Indeed, prior work has shown that *codY* is essential in *Streptococcus pneumoniae* and that a *codY* mutant strain carrying a *glnR* suppressor is highly susceptible to β-lactams.^80^

### Limitations of the study

Mutations in RNAP are among the most common adaptive mutations, but inferring a mechanism is challenging. The use of transcriptomics provides a view of how RNAP mutations may affect global gene expression. However, determining whether specific changes are adaptive, maladaptive, or neutral is not trivial, and resistance to one antibiotic can lead to collateral changes in sensitivity to other antibiotics.^33^ The genetic and physiological results reported here reveal a key role of the PyrR and CodY regulons in resistance to antibiotics that target class A PBPs. It is not yet clear why these effects are specific to class A PBPs. The role of BCAAs and CTP in membrane synthesis suggests an attractive model to link the observed pathway perturbations to β-lactam resistance. Indeed, previous results have revealed that downregulation of membrane synthesis (either genetically or by chemical inhibition) can restore growth to cells limited for PG synthesis and thereby suppress CEF^S^.^57^ However, further metabolomics and fluxomics studies will be needed to test this model. More generally, why altering Pyr metabolism affects β-lactam sensitivity is not yet resolved, and the mechanisms may vary between organisms. In methicillin-resistant *S. aureus*, RNAP mutations that increase Pyr nucleotides are thought to drive the synthesis of UDP-linked amino sugar precursors of PG, resulting in thickening of the PG layer and oxacillin resistance.^73^ In contrast, an *rpoB* mutation in *B. subtilis* that elevates UDP-GlcNAc levels confers CEF^S^ by downregulating PG synthesis through the GlmR pathway.^31^ These divergent results highlight the complexities inherent in understanding the effects of metabolic remodeling in the presence of antibiotics and how this can be altered by highly pleiotropic mutations such as those in RNAP.

## RESOURCE AVAILABILITY

### Lead contact

Further information and requests for resources and reagents should be directed to and will be fulfilled by the lead contact, John D. Helmann (jdh9@cornell.edu).

### Materials availability

Primary materials generated during this study are available upon request from the lead contact.

### Data and code availability

- All genome-aligned RNA-seq data described in this paper have been deposited into the NCBI GEO database and Cornell eCommons. These datasets are publicly available as of the date of publication. Accession numbers are listed in the key resources table.
- This paper does not report original code.
- Any additional information required to reanalyze the data reported in this paper is available from the lead contact upon request.

## STAR★METHODS

### EXPERIMENTAL MODEL AND STUDY PARTICIPANT DETAILS

All strains used in this study have been mentioned in key resources table. To construct deletion mutants, the BKE gene deletion collection^81^ was ordered from National Institute of Genetics, Microbial Physiology Laboratory, NBRP *B. subtilis*. These gene deletions were then transformed into the desired strains. After any genetic manipulations, the *rpoB* and *rpoC* mutations were always confirmed by Sanger sequencing at the Cornell Institute of Biotechnology. To remove the antibiotic cassette the cells were transformed with pDR244.^81,82^ Gene deletions were confirmed using check primers mentioned in key resources table. Genes were ectopically expressed at the *amyE* locus under promoter P*_hyspac_* using pPL82 plasmid.^83^ The *codY* and *pyrR* genes were cloned in pPL82 using the primers mentioned in key resources table.

### METHOD DETAILS

#### Growth conditions

For culturing, the glycerol stocks were streaked on lysogeny broth (LB) agar plates and incubated overnight at 37°C. For mid-log phase cultures, colonies were inoculated in 5 mL of LB and incubated at 37°C with shaking at 200 rpm till the cells reached ~0.4–0.5 OD_600nm_. For growth in minimal media (MMG), the following media composition was used: 2 mg/mL (NH_4_)_2_SO_4_, 0.2 mg/mL MgSO_4_.7H_2_O, 1 mg/mL potassium glutamate, 40 mM MOPS (pH 7.4), 5 mM potassium phosphate buffer (pH 7.0), 0.01 mg/mL tryptophan, 2% glucose, 5 μM FeSO_4_, 5 μM MnCl_2_. For amino acid supplementation, 2X MMG was supplemented with the required amino acids and then the volume adjusted with sterile Milli-Q water to make 1X media. For transformation, natural competency was induced in the cells by growing them till ~0.8 OD_600nm_ in modified competence (MC) media. 15 μg/mL kanamycin, combination of 1 μg/mL of erythromycin and 25 μg/mL lincomycin (mLs),100 μg/mL of spectinomycin, or 10 μg/mL of chloramphenicol was used to screen the transformants.

#### Suppressor analysis

0.4 OD_600nm_ *rpoB* H482Y mutant cells were plated on LB agar plates with a disc containing 25 μg of CEF placed in the center. CEF^R^ suppressors were picked up from the clear zone of inhibition. For RIF resistant suppressors, *rpoC* G1122D mutant cells were plated on 25, 50, 100 and 200 μg/mL RIF. Colonies were picked up from agar plates containing 25 and 50 μg/mL of RIF. Suppressors were tested for their MICs and resistant colonies were sent out for whole genome sequencing to SeqCenter (Pittsburgh). The reads were processed and analyzed with CLC Genomics workbench using NC_000964 as a reference genome to identify any single nucleotide or multi nucleotide variations.

#### Growth measurements

1 μL of ~0.4–0.5 OD_600nm_ cells were inoculated in 199 μL of media with the desired conditions. For ectopic induction of *codY* and *pyrR*, 1 mM isopropyl β-D-1-thiogalactopyranoside (IPTG) was supplemented in the media. The growth was monitored every 15 min for 24 h at 37°C in Bioscreen C Pro. All data was collected with at least 3 biological replicates and is plotted on a log_2_ axis as an average of the 3 replicates with the standard deviation represented as shaded region.

#### Minimum inhibitory concentrations (MICs)

Since *B. subtilis* is a non-pathogenic bacterium, there are no recommended Clinical & Laboratory Standards Institute (CLSI) guidelines for antibiotic susceptibility testing (AST), and we use the terms resistance and susceptible only to refer to increases and decreases in MIC relative to our wild-type parent strain. For RIF, MIC was performed by broth dilution method. 1 μL of ~0.4 OD_600nm_ (~5 × 10^5^ cells/mL) cells were inoculated in 199 μL of LB media with a 2-fold increasing dilution series from 0.0075 to 2 mg/mL of RIF. The plate was incubated with continuous shaking at 37°C in Bioscreen C Pro. The minimum concentration at which there was no growth after 12 h of treatment was determined as the MIC. The experiments were done in three biological replicates and MIC is reported as an average of the replicates. For CEF, the MIC was determined using E-test strips of concentration range 0.016–256 μg/mL (Liofilchem, Cat. No. 92129). 100 μL of ~0. 4 OD_600nm_ were mixed with 4 mL of LB soft agar (0.75% agar) and plated on 15 mL LB agar plates. The plates were allowed to air-dry for 15 min. An E-test strip was placed in the center of the plate and the plates were incubated for 18–20 h at 37°C. These experiments were done in at least three biological replicates and the MIC was reported as an average with the standard deviation. The average values were used to calculate the fold change in the MICs by dividing the higher MIC with the lower MIC value.

#### RNA sequencing

RNA was isolated from 1.5 mL of cells grown till ~0.4 OD_600nm_ using QIAGEN RNeasy Kit (Cat. No. 74106). Genomic DNA contamination was removed by treating RNA with TURBO DNA-free kit from Invitrogen (Cat. No. AM1907). The concentration and quality of the RNA sample was confirmed by Nanodrop. These samples were then submitted to the transcriptional regulation and expression facility (TREx) at Cornell University. The RNA integrity was determined by fragment analyzer (Agilent). All samples had an RQN value > 9. Total RNA samples were treated with NEBNext rRNA depletion kit for ribosomal RNA subtraction. UDI-barcoded RNA-seq libraries were generated with the NEBNext Ultra II RNA Library Prep Kit. Each library was quantified with Qubit (dsDNA HS kit; Thermo Fisher) and the size distribution was determined with Fragment Analyzer prior to pooling. Libraries were sequenced on NovaSeq 6000 (Illumina). At least 10M reads were generated per library.

#### RNA-seq data analysis

Reads were trimmed for low quality and adaptor sequences with TrimGalore v0.6.0, a wrapper for cutadapt and fastQC. Unwanted reads were removed with STAR v 2.7.0e. Reads were mapped to the reference genome (ensemble database, *Bacillus subtilis* subsp. *subtilis* str. 168 (GCA_000009045)) using STAR v2.7.0e. SARTools and DESeq2 v1.26.0 were used to generate normalized counts and statistical analysis of differential gene expression. The raw and analyzed RNA-seq data files have been deposited in the Cornell repository. The link to the data files has been mentioned in the key resources table.

#### Real-time PCR

RNA isolation and removal of genomic DNA contamination was done as described for RNA-seq. The DNase treated RNA was quantified using Nanodrop. 2 μg of RNA was reverse transcribed using High-Capacity cDNA Reverse Transcription Kit (Applied Biosystems, Cat. No. 4368814). The cDNA was diluted 1:10 to obtain 10 ng cDNA/μL. 1 μL of cDNA was used per reaction with 1x SYBR Master mix (Applied Biosystems, Cat. No. A25742) and 0.5 μM gene specific primers. QuantStudio 7 Pro (ThermoFisher Scientific) at the Cornell Institute of Biotechnology was used for detecting the transcript levels. *gyrA* was used as an internal control. Gene expression values were calculated as 2^−ΔC^_T_ after normalization with *gyrA*. The data was plotted on log_10_ scale. RNA was isolated from three biological replicates and gene expression of each biological replicate has been represented as a dot in the graph.

#### Reactive radical species (RRS) detection

Cells were grown up to ~0.4 OD_600nm_. 500 μL of the cells were immediately mixed with 500 μL of LB with 10 μg/mL of DCFDA with and without 20.48 μg/mL CEF and incubated for 1 h at 37°C with shaking. After 1 h, the cells were centrifuged at 8000 rpm for 5 min and resuspended in cold phosphate buffer saline (PBS). 200 μL of these cells were aliquoted in clear bottom black 96-well plates. Fluorescence (ex. 488 nm, em. 520 nm) and OD_600nm_ was measured in Synergy H1 microplate reader (Biotek). Data was collected for 4 biological replicates and plotted as a ratio of Fluorescence/OD_600nm_ of CEF treated by untreated cells.

### QUANTIFICATION AND STATISTICAL ANALYSIS

Number of replicates used for each experiement, the statistical analysis methods, and *p*-value cut-offs used to define significance were all indicated in the figure legends and method details. All data were analyzed using the GraphPad Prism version 10.

## Supplementary Material

1

## Figures and Tables

**KEY RESOURCES TABLE:** 

REAGENT or RESOURCE	SOURCE	IDENTIFIER

Bacterial and virus strains		

HB26336	Wild-type (WT) 168	Lab stock
HB26341	*rpoB* C1444T (RNAP β-subunit H482Y)	From study (Patel et al.^31^)
HB26291	*rpoC* G3365A (RNAP β'-subunit G1122D)	From study (Lee et al.^29^), kan^R^
HB28141	*rpoB* A1445G (RNAP β-subunit H482Q)	Created by transformation of *rpoB* mutant gene into WT
HB26332	*rpoB* A1445G *rpoC* G3365A (RNAP β subunit H482Q - β' subunit G1122D)	RIF suppressor in *rpoC* G1122D strain
HB25950	*ponA*::*erm* (Δ*ponA*)	From study (Patel et al.^35^)
HB28144	*rpoC* G1122D:*kan ponA*::*erm*	HB25950 transformed in HB26291
HB28305	*codY*::*erm*	BKE16170 transformed in HB26336
HB28306	*codY* null (Δ*codY*)	Cassette removed from HB28305 using pDR244
HB28314	Δ*codY rpoC* G1122D:*kan*	*rpoC* G1122D transformed in HB28306
HB28604	*amyE*::P_*hyspac*_-*codY*	pPL82 construct with *codY* transformed in HB26336
HB28334	*relA*::*erm*	BKE27600 transformed in HB26336
HB28461	*sigD*::*erm*	BKE16470 transformed in HB26336
HB28458	*lytC*::*erm*	BKE35620 transformed in HB26336
HB28452	*lytD*::*erm*	BKE35780 transformed in HB26336
HB28443	*lytF*::*erm*	BKE09370 transformed in HB26336
HB28301	*pyrR*::*erm*	BKE15470 transformed in HB26336
HB28302	*pyrR* null (Δ*pyrR*)	Cassette removed from HB28302 using pDR244
HB28309	Δ*pyrR rpoC* G1122D:*kan*	*rpoC* G1122D transformed in HB28302
HB28317	Δ*pyrR codY*::*erm*	HB28305 transformed in HB28302
HB28320	Δ*pyrR*Δ*codY*	Cassette removed from HB28317 using pDR244
HB28380	Δ*pyrR*Δ*codY rpoC* G1122D:*kan*	*rpoC* G1122D transformed in HB28320
HB28607	*amyE*::P_*hyspac*_*-pyrR*	pPL82 construct with pyrR transformed in HB26336
HB28007	*glmR*::*erm*	BKE34760 transformed in HB26336
HB28628	*amyE*::P_*hyspac*_*-pyrR-glmR*::*erm*	HB28007 transformed in HB28607
HB28615	*ptsG*::*kan*	BKK13890 transformed in HB26336
HB28596	*sacP*::*erm*	BKE38050 transformed in HB26336
HB28597	*fruA*::*erm*	BKE14400 transformed in HB26336
HB28617	*qoxA*::*erm*	BKE38170 transformed in HB26336

Deposited data		

RNA-seq raw and analyzed data	NCBI GEO database Cornell eCommons	accession series: GSE285388

Oligonucleotides		

rpoB-FP	GATGAAGTTTCCGTCGTTCAA	To amplify *rpoB*
rpoB-RP	GAAAATGCGTTCGCAGAATAG	To amplify *rpoB*
rpoB-seq	CTTCTCCAGAACCAATTCCGT	To sequence the mutation at 1444/5 position of *rpoB*
rpoC-FP	TATCACACAGGGTCTTCCGC	To amplify part of *rpoC*
rpoC-RP	AAGCAGTAACCTCGATTCCGT	To amplify part of *rpoC*
rpoC-seq	TTCTCCATGAGGTTCAAAAGG	To sequence the mutation at 3365 position of *rpoC*
ilvD-RT-FP	CGTCACTTGTTCTTCCTGCC	For detection by qRT PCR
ilvD-RT-RP	ACTGCGACAAAATCACACCG	For detection by qRT PCR
ilvK-RT-FP	TCCCGCAAATTGATGAAGAACA	For detection by qRT PCR
ilvK-RT-RP	CGATGATGAACGGACGGATG	For detection by qRT PCR
mtnA-RT-FP	GAATGGCTTCATGGACAAGGT	For detection by qRT PCR
mtnA-RT-RP	GCCTACCGCCATTAATTTGTCA	For detection by qRT PCR
mtnK-RT-FP	TCAAACGTTTCCCAGACCTG	For detection by qRT PCR
mtnK-RT-RP	GATCGGCTTTGATGTAGGGC	For detection by qRT PCR
pyrAA-RT-FP	ATGTGAGCTGCCTTCCAACT	For detection by qRT PCR
pyrAA-RT-RP	CGTGCGGATCATTCTTGTCA	For detection by qRT PCR
pyrC-RT-FP	AAGCGTTAAAAGAAGCCGGG	For detection by qRT PCR
pyrC-RT-RP	GCTTTGTCAATTGCGGCTG	For detection by qRT PCR
alaT-RT-FP	AGTCAGGCTTTGGATATTGCGA	For detection by qRT PCR
alaT-RT-RP	CTGCCAAAGAAACAAGCGCA	For detection by qRT PCR
gyrA-RT-FP	GGCGGCCATGCGTTATACAG	For detection by qRT PCR
gyrA-RT-RP	GCCATACCTACCGCAATGCC	For detection by qRT PCR
sigD-RT-FP	CTCGGAATGACGGTACAGGA	For detection by qRT PCR
sigD-RT-RP	ACTTGAATGTTTTCCCCGTCA	For detection by qRT PCR
codY-check-FP	TGTCGTTTGAAGCTCCAGATG	To confirm *codY* deletion
codY-check-RP	AATATCCGCTCTGCTCAAGG	To confirm *codY* deletion
pyrR-check-FP	TATGTCGAATTTGAAGCGCCG	To confirm *pyrR* deletion
pyrR-check-RP	GTACGCCAGTGTTCCGATG	To confirm *pyrR* deletion
ponA-check-FP	CAAGACCTCTTTCCCCCTGC	To confirm *ponA* deletion
ponA-check-RP	CTGCACGGAATTACAAGGCG	To confirm *ponA* deletion
codY_SmaI_FP	TCCCCCGGGGGATTGTCGTTTGAAGCTCCAGATG	To clone codY in pPL82
codY_SphI_RP	ACATGCATGCATGTCTCGCCTTGATATAAGCCTGA	To clone codY in pPL82
pyrR_SmaI_FP	TCCCCCGGGGGAGGCAGAATTAATCGAAAACCTCA	To clone pyrR in pPL82
pyrR_SphI_RP	ACATGCATGCATGCTGAAGGCTGAATGAAACCCA	To clone pyrR in pPL82
relA-check-FP	GGCCGCGTTCATGTAGTT	To confirm *relA* deletion
relA-check-RP	ATTAAGCCTGGTCAGCGTGT	To confirm *relA* deletion
sigD-check-FP	CCGGCGGATCAGAGATGTTT	To confirm *sigD* deletion
sigD-check-RP	CCGACGGTCAACCTCTGTAA	To confirm *sigD* deletion
lytC-check-FP	TCCAATGAGCGCAAACCAAA	To confirm *lytC* deletion
lytC-check-RP	GAACGACACAAGGGCAGC	To confirm *lytC* deletion
lytD-check-FP	TGCAAGTCACATTAAATGAACGC	To confirm *lytD* deletion
lytD-check-RP	GCAGCGTGATAGAAGGAAGC	To confirm *lytD* deletion
lytF-check-FP	CGAGTCCGTTCAAAAGTGGG	To confirm *lytF* deletion
lytF-check-RP	GCATGGACCCTGAAGACATC	To confirm *lytF* deletion
glmR-check-FP	CGAATTTCTCATGTCCCGAA	To confirm *glmR* deletion
glmR-check-RP	GATTGAAGCGCTGGATGAAA	To confirm *glmR* deletion
ptsG-check-FP	AGGCATGAGTGATTGAGGGA	To confirm *ptsG* deletion
ptsG-check-RP	GCGTCGTATTTGCTAGCAGT	To confirm *ptsG* deletion
sacP-check-FP	CCATTCGGGTCATTCAGCAG	To confirm *sacP* deletion
sacP-check-RP	TGAATAAGCGGGATTGTGACTG	To confirm *sacP* deletion
fruA-check-FP	GAATTTCCAAACAGCTGCCG	To confirm *fruA* deletion
fruA-check-RP	TCGCTTTACCCCATTCCAGG	To confirm *fruA* deletion
qoxA-check-FP	TCATGATTGTACCGTGAGTTGT	To confirm *qoxA* deletion
qoxA-check-RP	GGTAACCGTGTGAAAAGATGCT	To confirm *qoxA* deletion

## Highlights

- Mutations in RNAP that confer antibiotic resistance have collateral effects
- Remodeling of the transcriptome alters central metabolism
- Repression of BCAA and Pyr pathways contributes to β-lactam resistance
- β-lactam treatment leads to metabolic dysregulation linked to ROS production
