# Supplementary material for: A mutation in RNA polymerase imparts resistance to β-lactams by preventing dysregulation of amino acid and nucleotide metabolism
Source: Cell Rep. Author manuscript; Available in PMC 2025 Apr 7. (PMC11975431; doi:10.1016/j.celrep.2025.115268)
Supplement: 1 [file NIHMS2060931-supplement-1.pdf]

Cell Reports, Volume *44*

Supplemental information

A mutation in RNA polymerase imparts resistance to β-lactams by preventing dysregulation of amino acid and nucleotide metabolism

Yesha Patel and John D. Helmann

Figure S1

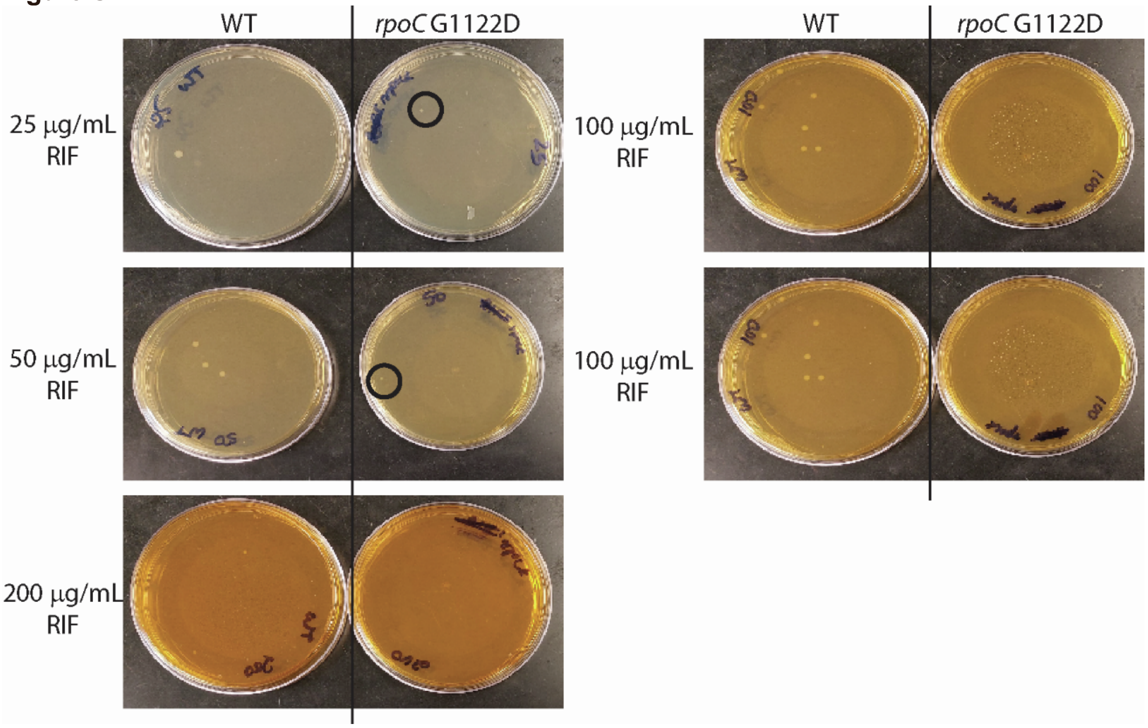

Figure S1 Plate images showing emergence of rifampicin (RIF) suppressors in WT and *rpoC* G1122D mutant. Suppressors were selected by growth on 25, 50, 100 and 200 μg/mL of RIF. The *rpoC* G1122D mutant had a comparatively lower frequency of acquiring mutations imparting resistance to RIF. The circled black colonies are the only 2 colonies from this forward selection on RIF.

Figure S2

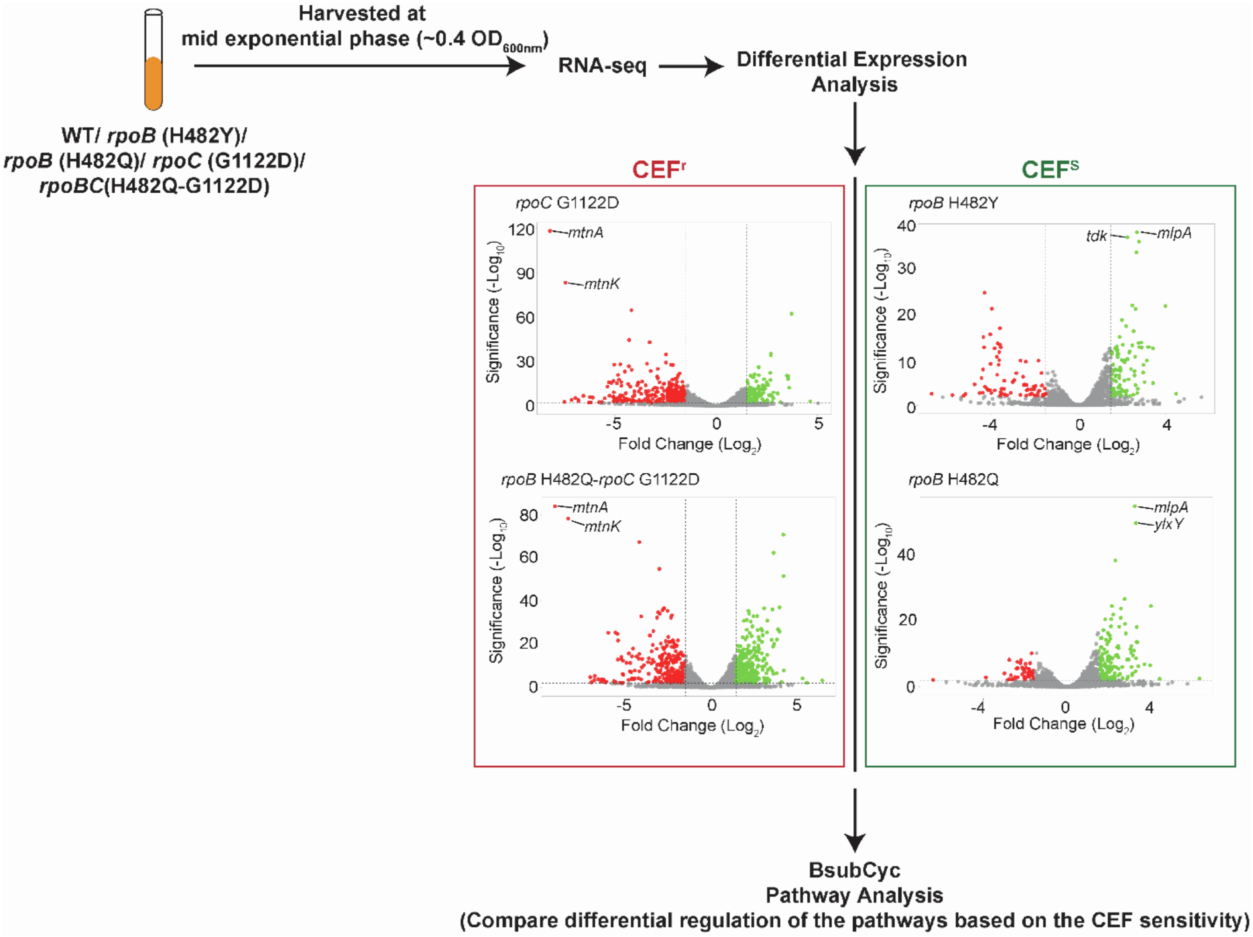

Figure S2 Schematic representation of the RNA-seq workflow. WT (HB26336), *rpoB* H482Y (HB26341), *rpoB* H482Q (HB28141), *rpoC* G1122D (HB26291) and *rpoB* H482Q-*rpoC* G1122D (HB26332) mutants were grown in LB medium till ~0.4 OD_{600nm}. RNA was isolated and sent for RNA-seq analysis. N=2. Average value of the reads was used in the analysis. Log_2 fold-change (log_2FC) and *p*-values were calculated for each mutant compared to the WT cells. The differentially expressed genes were visualized using volcano plots to identify genes that were significantly reduced in expression (red), significantly upregulated (green), or with no significant change (grey). Significance was based on the criteria of log_2FC of < -1.5 and > 1.5, and a $-log_{10}$ *p*-value > 2. The top two significant hits of each mutant are labelled. The significantly altered genes were analyzed using BsubCyc to identify pathways with a high perturbation score (PPS; arXiv:1510.03964).

Figure S3

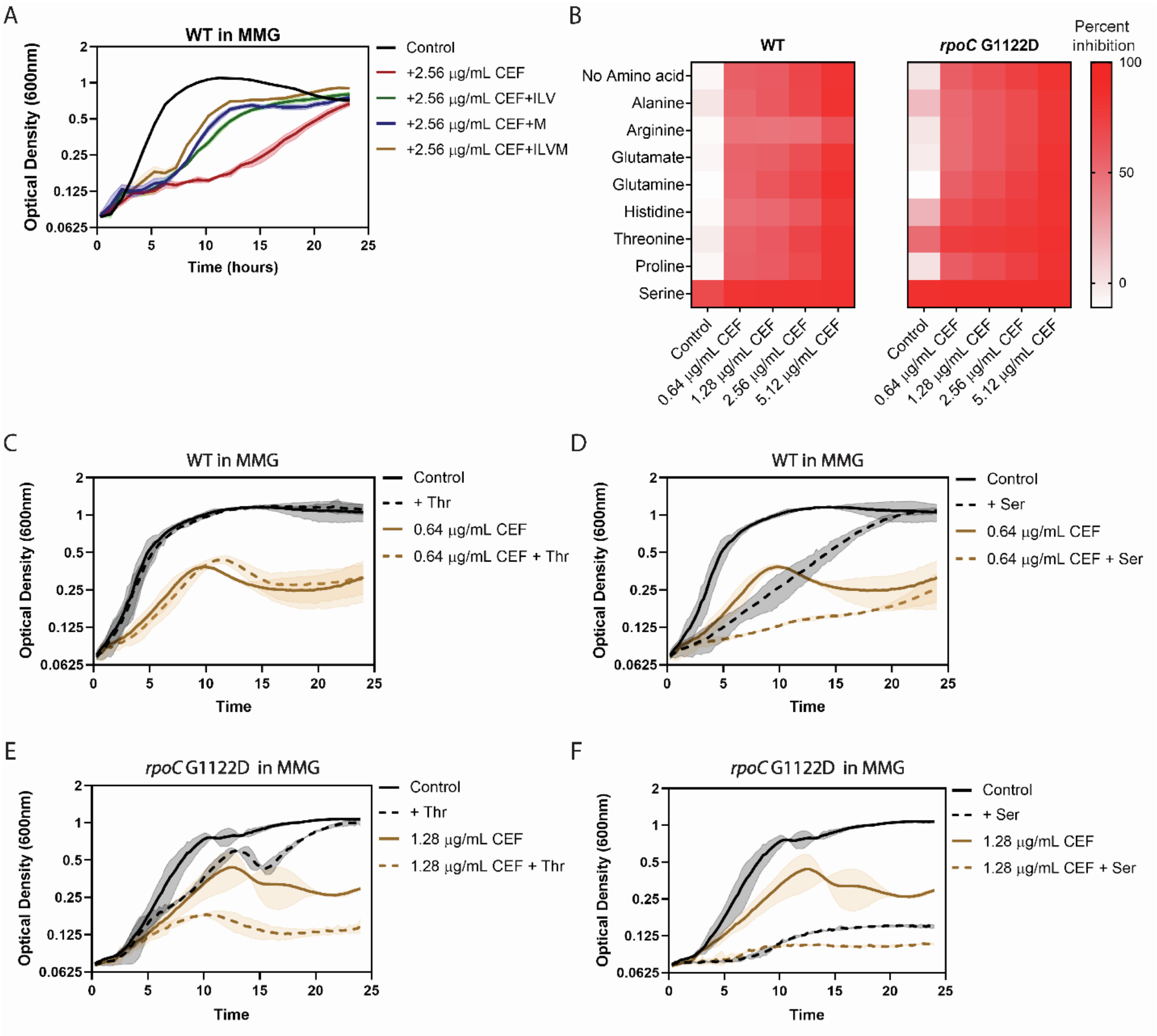

Figure S3 Effect of amino acid supplementation on CEF susceptibility (A) Growth of WT (HB26336) in MMG supplemented with the combination of 2 mg/mL of Ile, Leu, Val (ILV) and/or Met (M) in the presence of 2.56 µg/mL CEF. (B)) Heat map illustrating the percent inhibition of WT (HB26336) and *rpoC* G1122D (HB26291) mutant after 10 hours of treatment with 0, 0.64, 1.28, 2.56, or 5.12 µg/mL CEF in MMG with 2 mg/mL supplementation of the individual amino acids. (C) Growth of WT cells on treatment with 0.64 µg/mL CEF in MMG in the presence of 2 mg/mL of Threonine (Thr) (D) Growth of WT cells on treatment with 0.64 µg/mL CEF in MMG in the presence of 2 mg/mL of Serine (Ser) (E) Growth of *rpoC* G1122D mutant on treatment with 1.28 µg/mL CEF in MMG in the presence of 2 mg/mL of Thr (F) Growth of *rpoC* G1122D mutant on treatment with 1.28 µg/mL CEF in MMG in the presence of 2 mg/mL of Ser. Thr is a precursor for Ile biosynthesis, and high levels of Thr are proposed to generate toxic levels of 2-oxobutyrate that can inhibit Val synthesis. The high sensitivity of the *rpoC* mutant to Thr-mediated growth inhibition may be due to this same mechanism exacerbated by the altered regulation of the BCAA pathways, or possibly to another mechanism altogether. Consistently, the *rpoC* mutant was also found to have reduced expression of Thr uptake and biosynthesis genes (Table S3).

Figure S4

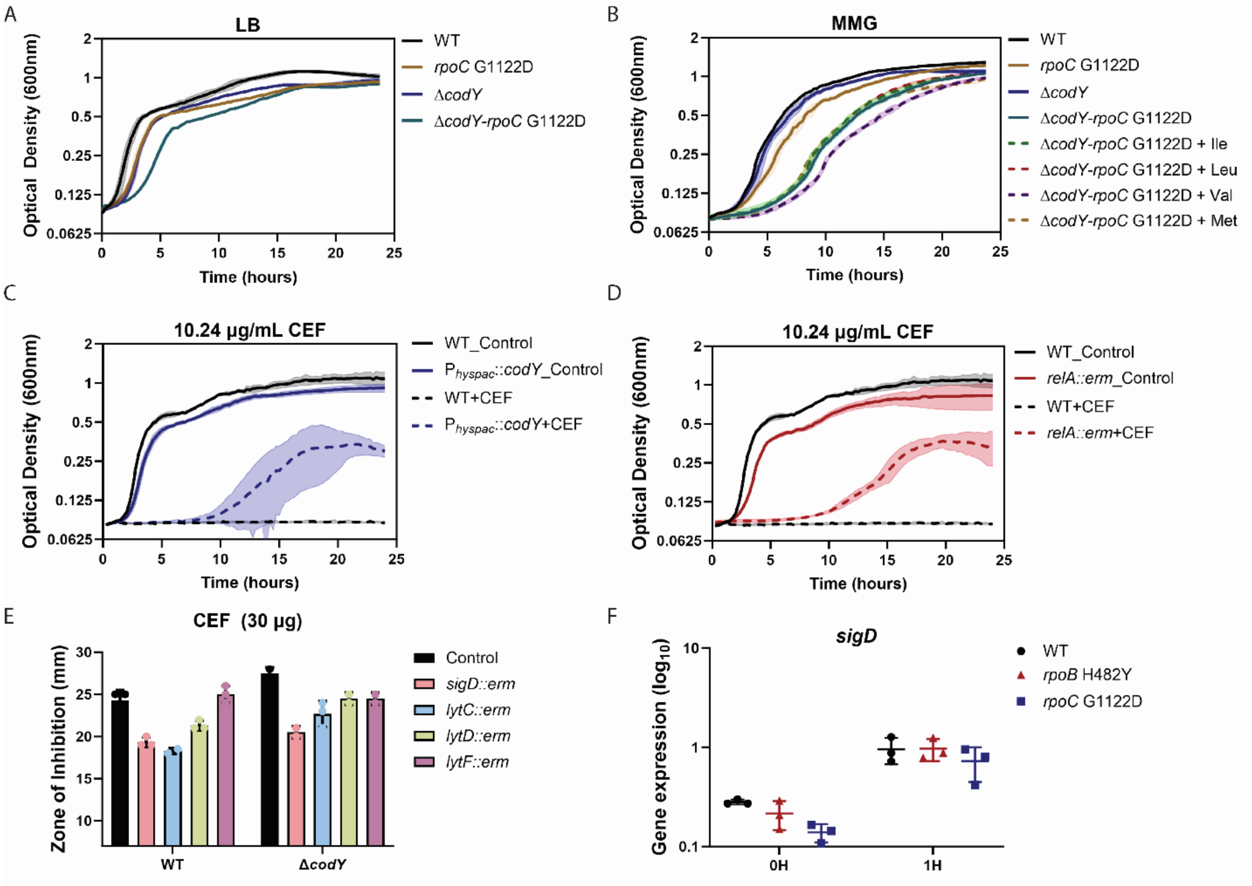

Figure S4 Effect of CodY on CEF susceptibility. Growth of WT (HB26336), *rpoC* G1122D (HB26291), Δ*codY* (HB28306), and Δ*codY-rpoC* G1122D (HB28314) double mutant in (A) LB or (B) MMG medium. The growth of the Δ*codY-rpoC* G1122D double mutant on supplementation with 2 mg/mL of isoleucine (Ile), leucine (Leu), valine (Val) and methionine (Met) independently. (C) Growth of WT cells with and without induction of *codY* from P_{hyspac} promoter integrated at the *amyE* locus (HB28604). LB was supplemented with 10.24 µg/mLCEF and 1 mM IPTG for induction of CodY. (D) Growth of *relA::erm* (HB28334) mutant cells in LB supplemented with 10.24 µg/mL CEF. (E) CEF^S measured by zone of inhibition for WT (HB26336), *sigD*::*erm* (HB28461), *lytC*::*erm* (HB28458), *lytD*::*erm* (HB28452), *lytF*::*erm* (HB28443) strains. Discs with 30 µg of CEF were used for this assay (BD Biosciences, Cat # 231621). (F) *sigD* levels were measured in WT (HB26336), *rpoB* H482Y (HB26341) and *rpoC* G1122D (HB26291) mutant by real-time PCR before and after 1 hour treatment with 0.64 µg/mL of CEF. Gene expression values are plotted on a log_{10} scale after normalization to *gyrA* as internal control. N=3. Each dot represents the individual value.

Figure S5

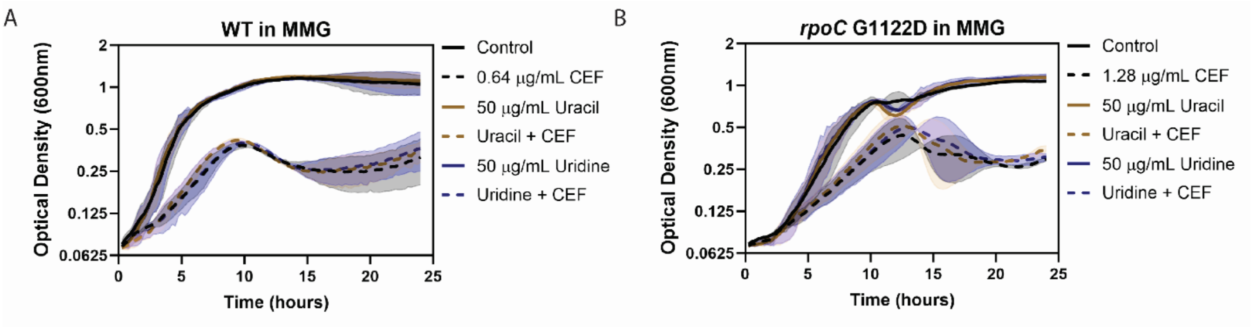

Figure S5 Supplementation of pyrimidines. (A) Growth of WT (HB26336) cells in MMG on supplementation with 50 µµg/mL of uracil or uridine, without and with 0.64 µg/mL CEF. (B) Growth of *rpoC* G1122D (HB26291) mutant in MMG on supplementation with 50 µg/mL of uracil or uridine, without and with 0.64 µg/mL CEF.

Figure S6

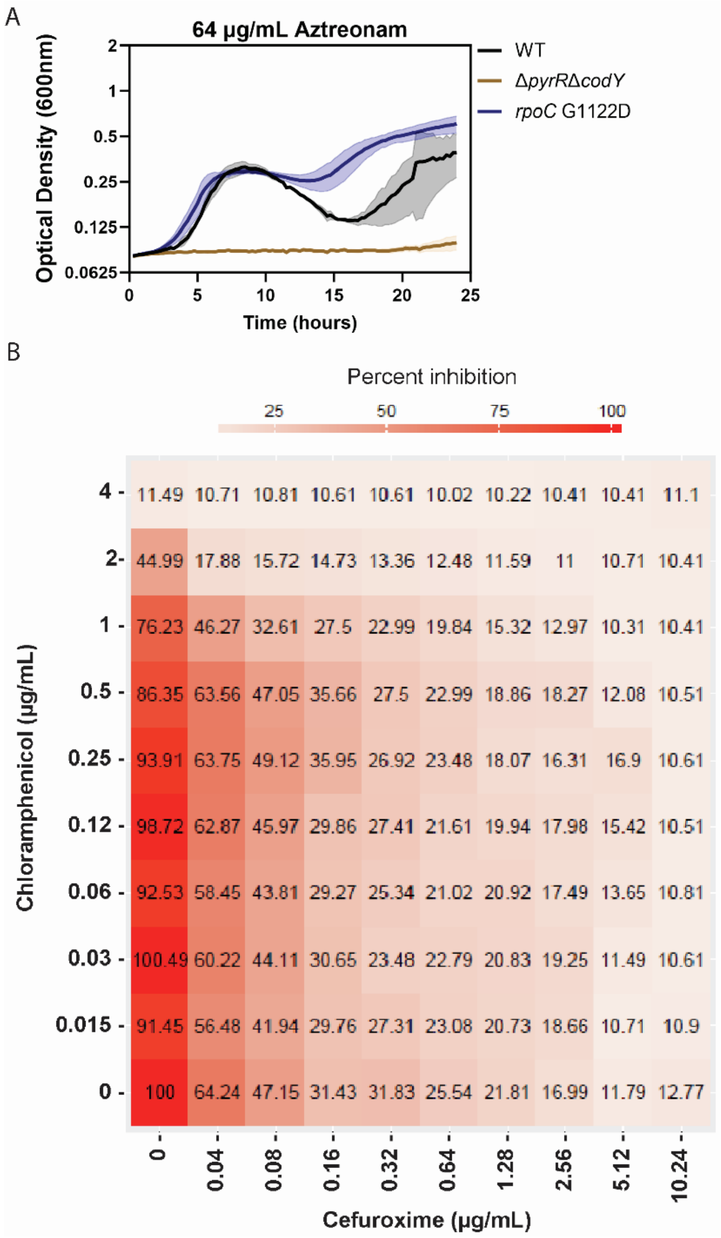

Figure S6 Effect of other drugs (A) Growth of WT (HB26336), Δ*pyrR*Δ*codY* (HB28320) and *rpoC* G1122D (HB26291) mutant in the presence of 64 µg/mL of aztreonam (AZT) (B) Representation of the checkerboard assay to determine the interaction between chloramphenicol, a protein synthesis inhibitor, and CEF. Data plotted as percent inhibition after 10 hours of treatment with individual drugs as well as drug combinations.

Figure S7

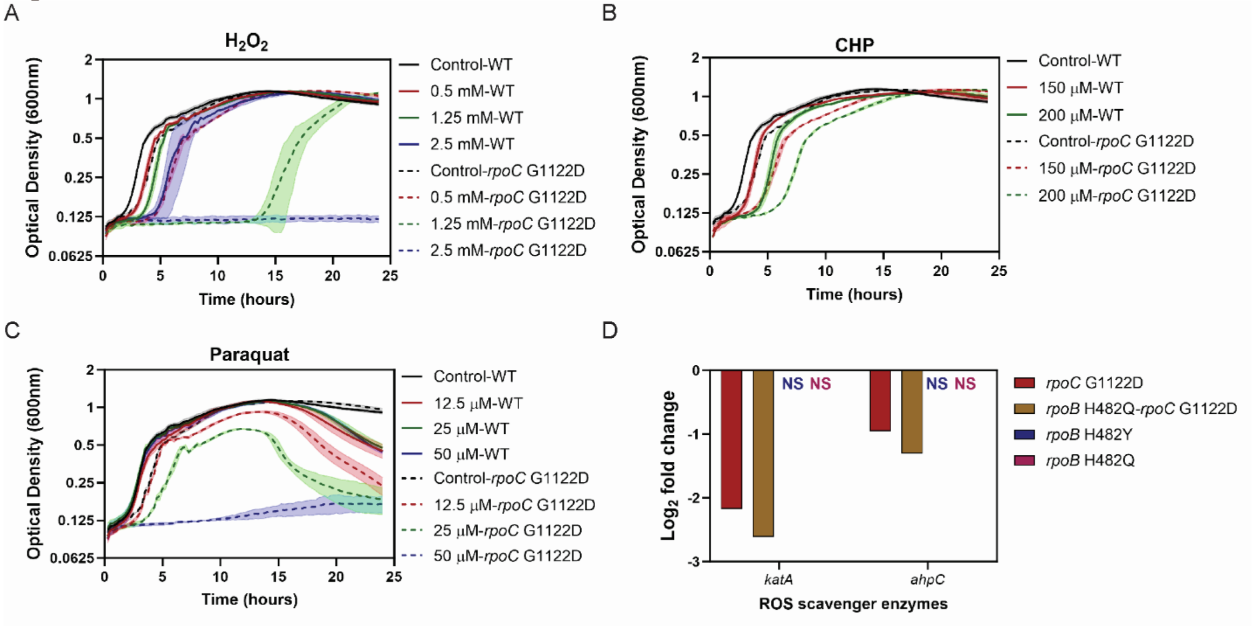

Figure S7 Effect of oxidants. (A-C) Growth kinetics of WT (HB26336) and *rpoC* G1122D (HB26291) mutant upon treatment with varying concentrations of oxidants: (A) H_2O_2, (B) cumene hydroperoxide (CHP), and (C) paraquat. (D) Gene expression data of reactive oxygen species (ROS) scavenger enzymes (*katA* and *ahpC*) in *rpoC* G1122D (HB26291), *rpoB* H482Q-*rpoC* G1122D (HB26332), *rpoB* H482Y (HB26341) and *rpoB* H482Q (HB28141). Results obtained from the RNA-seq experiment are plotted as $\log_2$ fold change ratio compared to WT cells.

Table S1 CEF and RIF MICs of different strains used in this study (For RIF, MIC was calculated as the minimum concentration which had no growth in LB media after 12 hours of drug treatment, N=3. R indicates resistant compared to WT and S indicates sensitive compared to WT. For CEF, MIC was detected by E-test strip on LB agar plates, N= atleast 3). The significance between the mics of mutants and WT was calculated using students's ttest (ns indicates P > 0.05, * indicates P ≤ 0.05, ** indicates P ≤ 0.01, *** indicates P ≤ 0.001, **** indicates P ≤ 0.0001).

**Strains**	**Nucleotide change**	**CEF MIC (µg/mL)**	**Fold-change**	**RIF MIC (µg/mL)**	**Fold-change**
WT		2.3 ± 0.7	-	0.2 ± 0.7	-
β H482Y	*rpoB* C1444T	0.05 ± 0.01***	46 (S)	>2	>10 (R)
β H482Q	*rpoB* A1445G	0.05 ± 0.01***	46 (S)	>2	>10 (R)
β' G1122D	*rpoC* G3365A	17.6 ± 3.6****	8 (R)	0.06*	3 (S)
β H482Q –<br>β' G1122D	*rpoB* A1445G<br>*rpoC* G3365A	10 ± 2***	4 (R)	>2	>10 (R)
Δ*ponA*	*ponA::erm*	9.3 ± 2.3***	4 (R)	not tested	
Δ*ponA*<br>β' G1122D	*ponA::erm*<br>*rpoC* G3365A	29.3 ± 4.6****	13 (R)	not tested	
Δ*codY*	*codY null*	1.3 ± 0.3^{ns}	2 (S)	not tested	
Δ*codY*<br>β' G1122D	*codY null*<br>*rpoC* G3365A	16 ± 0****	7 (R)	not tested	
Δ*pyrR*	*pyrR null*	0.7 ± 0.1**	3 (S)	not tested	
Δ*pyrR*<br>β' G1122D	*pyrR null*<br>*rpoC* G3365A	18.7 ± 4.6***	8 (R)	not tested	
Δ*pyrR*Δ*codY*	*codY null*<br>*pyrR null*	0.08 ± 0.02**	27 (S)	not tested	
Δ*pyrR*Δ*codY*<br>β' G1122D	*codY null*<br>*pyrR null*<br>*rpoC* G3365A	8.7 ± 3.1**	4 (R)	not tested	

Table S2 Gene expression values (log_2 fold-change) of UMP biosynthesis pathway, L-methionine salvage cycle, BCAA uptake and metabolism. The differentially expressed genes were highlighted to indicate >2-fold decreased expression (red), ≤2-fold decreased expression (pink), increased expression (green), or no significant change (grey) in the indicated mutant strain relative to WT.

**Gene**	**Description**	***rpoC* G1122D**	***rpoB* H482Y-*rpoC* G1122D**	***rpoB* H482Y**	***rpoB* H482Q**
**UMP biosynthesis**					
pyrAA	pyrimidine-specific carbamoyl-phosphate synthetase	-5.1	-5.2	NS	NS
pyrAB	pyrimidine-specific carbamoyl-phosphate synthetase	-4.7	-4.8	NS	NS
pyrB	aspartate carbamoyltransferase	-3.9	-4.5	NS	NS
pyrC	dihydroorotase	-4.5	-4.7	NS	NS
pyrK	dihydroorotate dehydrogenase (electron transfer subunit)	-4.6	-4.7	NS	NS
pyrD	dihydroorotate dehydrogenase (catalytic subunit)	-4.2	-4.3	NS	NS
pyrE	orotate phosphoribosyltransferase	-4.7	-4.4	NS	NS
pyrF	orotidine 5'-phosphate decarboxylase	-4.3	-4.1	NS	NS
**L- Methionine salvage cycle**					
mtnU	ketoglutaramate omega-amidase	-2.9	-2.4	NS	NS
mtnE	methionine-glutamine aminotransferase	-2.9	-3.5	NS	NS
mtnD	acireductone dioxygenase	-2.3	-1.2	NS	NS
mtnX	2-hydroxy-3-keto-5-methylthiopentenyl-1-phosphate phosphatase	-2.7	-1.7	NS	NS
mtnW	2,3-diketo-5-methylthiopentyl-1-phosphate enolase	-2.4	-1.9	NS	NS
mtnB	methylthioribulose-1-phosphate dehydratase	-2.8	-1.6	NS	NS
mtnA	methylthioribose-1-phosphate isomerase	-8.2	-9.2	-0.8	NS
mtnK	methylthioribose kinase	-7.4	-8.5	NS	NS
mtnN	methylthioadenosine / S-adenosylhomocysteine nucleosidase	NS	NS	-0.8	NS
speE	spermidine synthase	NS	NS	NS	NS
speD	S-adenosylmethionine decarboxylase	NS	-0.7	NS	NS
metK	S-adenosylmethionine synthetase	0.7	NS	NS	NS
**Methionine Uptake**					
metN	methionine ABC transporter (ATP-binding protein)	-1.7	-3.1	-0.6	NS
metP	methionine ABC transporter, permease component	-2.0	-3.2	-0.8	NS
metQ	methionine ABC transporter, substrate binding lipoprotein	-2.0	-2.9	-0.7	NS

**Branched chain amino acid metabolism**					
ilvA	threonine dehydratase	**-1.2**	**-1.1**	NS	NS
ilvH	acetohydroxy-acid synthase (small subunit)	**-4.5**	**-4.3**	NS	NS
ilvB	acetohydroxy-acid synthase (large subunit)	**-4.8**	**-6.1**	**-1.4**	NS
alsS	alpha-acetolactate synthase	**1.7**	**1.3**	0.6	NS
ilvC	acetohydroxy-acid isomeroreductase	**-3.8**	**-4.0**	**-1.7**	NS
ilvD	dihydroxy-acid dehydratase	**-3.5**	**-3.3**	NS	NS
ilvK	branched-chain amino acid aminotransferase	**-2.5**	**-1.1**	**0.7**	**1.0**
ilvE	aromatic amino acid aminotransferase	**-1.2**	**-1.4**	NS	NS
leuA	2-isopropylmalate synthase	**-3.0**	**-3.2**	**-1.4**	NS
leuC	3-isopropylmalate dehydratase (large subunit)	**-2.0**	**-1.9**	**-1.3**	**-1.1**
leuD	3-isopropylmalate dehydratase (small subunit)	**-1.1**	**-1.8**	NS	**-1.0**
leuB	3-isopropylmalate dehydrogenase	**-2.1**	**-1.8**	**-1.2**	NS
**Branched chain amino acid uptake**					
bcaP	branched-chain amino acid transporter	**-2.2**	**-2.1**	NS	NS
braB	branched-chain amino acid-Na^+ symporter	**-1.3**	**-1.2**	NS	NS
brnQ	low-affinity branched-chain amino acid transporter	**0.5**	**0.6**	NS	NS

Table S3 Gene expression values (log_2 fold-change) of threonine biosynthesis and uptake. The differentially expressed genes were highlighted to indicate >2-fold decreased expression (red), ≤2-fold decreased expression (pink), increased expression (green), or no significant change (grey) in the indicated mutant strain relative to WT.

Gene	Description	*rpoC* G1122D	*rpoB* H482Y-*rpoC* G1122D	*rpoB* H482Y	*rpoB* H482Q
**Threonine biosynthesis**					
thrZ	threonyl-tRNA synthetase	**-2.9**	**-1.5**	**0.8**	**1.0**
thrC	threonine synthase	**-1.9**	**-2.3**	**-1.0**	**-0.9**
thrB	homoserine kinase	**-1.7**	**-2.2**	**-1.0**	NS
thrR	transcriptional repressor of operons *hom-thrCB* and *thrD*	NS	NS	NS	NS
thrD	aspartate kinase III	**-1.0**	**-0.9**	NS	NS
thrS	threonyl-tRNA synthetase	**-0.9**	**-0.7**	NS	NS
**Threonine uptake**					
bcaP	branched-chain amino acid transporter	**-2.2**	**-2.1**	NS	NS

Table S4 MICs of WT, Δ*pyrR*Δ*codY* and *rpoC* G1122D mutant against mecillinam and cloxacillin measured by E-test strip.

Strains	Cloxacillin MIC (μg/mL)	Mecillinam MIC (μg/mL)
WT	0.25 ± 0.0	1.2 ± 0.3
rpoC G1122D	0.25 ± 0.1	1.8 ± 0.3
Δ*pyrR*Δ*codY*	0.15 ± 0.04	1.8 ± 0.3

Table S5 Gene expression values (log_2 fold-change) of sugar-specific transporters, *cyd* operon and enzymes involved in nitrate respiration. The differentially expressed genes were highlighted to indicate >2-fold decreased expression (red), increased expression (green), or no significant change (grey) in the indicated mutant strain relative to WT.

Gene	Description	*rpoC* G1122D	*rpoB* H482Y-*rpoC* G1122D	*rpoB* H482Y	*rpoB* H482Q
**Sugar-specific transporters**					
mtlA	mannitol-specific permease	**-3.7**	**-1.4**	NS	NS
ptsG	glucose permease	**-2.6**	**-2.4**	NS	NS
fruA	fructose-specific permease	**-2.2**	**-1.1**	NS	NS
sacP	sucrose permease	**-1.8**	NS	NS	NS
manP	mannose-specific permease	3.6	3.2	-1.6	1.3
**Cyd operon**					
cydA	cytochrome *bd* ubiquinol oxidase	**-3.8**	**-2.4**	NS	NS
cydB	cytochrome *bd* ubiquinol oxidase	**-5.5**	NS	NS	NS
cydC	ABC transporter required for expression of cytochrome *bd*	**-6.1**	NS	NS	NS
cydD	ABC transporter required for expression of cytochrome *bd*	**-4.7**	NS	NS	NS
**Nitrate respiration**					
narG	nitrate reductase (alpha subunit)	**-7.0**	**-6.9**	NS	NS
narH	nitrate reductase (beta subunit)	**-6.8**	**-6.4**	NS	NS
narI	nitrate reductase (gamma subunit)	**-6.8**	**-6.2**	NS	NS
narJ	chaperone for the nitrate reductase	**-7.5**	**-7.1**	NS	NS
narK	nitrite extrusion protein	**-4.2**	**-3.9**	NS	NS
